# Defining diet quality: a synthesis of dietary quality metrics and their validity for the double burden of malnutrition

**DOI:** 10.1016/S2542-5196(20)30162-5

**Published:** 2020-08-12

**Authors:** Victoria Miller, Patrick Webb, Renata Micha, Dariush Mozaffarian, Global Dietary Database

**Affiliations:** a Friedman School of Nutrition Science and Policy, Tufts University, Boston, MA, USA

## Abstract

Achieving most of the UN Sustainable Development Goals requires a strong focus on addressing the double burden of malnutrition, which includes both diet-related maternal and child health (MCH) and non-communicable diseases (NCDs). Although, the most optimal dietary metric for assessing malnutrition remains unclear. Our aim was to review available global dietary quality metrics (hereafter referred to as dietary metrics) and evidence for their validity to assess MCH and NCD outcomes, both separately and together. A systematic search of PubMed was done to identify meta-analyses or narrative reviews evaluating validity of diet metrics in relation to nutrient adequacy or health outcomes. We identified seven dietary metrics aiming to address MCH and 12 for NCDs, no dietary metrics addressed both together. Four NCD dietary metrics (Mediterranean Diet Score, Alternative Healthy Eating Index, Healthy Eating Index, and Dietary Approaches to Stop Hypertension) had convincing evidence of protective associations with specific NCD outcomes, mainly mortality, cardiovascular disease, type 2 diabetes, and total cancer. The remaining NCD dietary metrics and all MCH dietary metrics were not convincingly validated against MCH or NCD health outcomes. None of the dietary metrics had been validated against both MCH and NCD outcomes. These findings highlight major gaps in assessing and addressing diet to achieve global targets and effective policy action.

## Introduction

Poor diet quality is a leading and preventable cause of adverse health globally, which includes both maternal and child health (MCH) and non-communicable diseases (NCDs).^1^, ^2^ The UN Sustainable Development Goals (SDGs) outline global consensus on social, economic, environmental, and health targets to be met by 2030, with most goals concerned with nutrition including one goal to end malnutrition.^3^ Yet rates of progress toward achieving the SDGs have been slow, and accelerated momentum is needed.^4^, ^5^, ^6^, ^7^, ^8^ To develop sound strategies and monitor progress toward these goals, the assessment of global dietary quality is essential.

Although a variety of dietary metrics have been developed and used to summarise various components of a diet (eg, adequacy, quality, diversity),^9^, ^10^ there remains an absence of widely used, validated metrics to define the double burden of malnutrition, and to compare effectively across country settings. This absence at least partly relates to a historical distinction between global nutrition efforts for MCH versus NCDs.^11^, ^12^, ^13^ Malnutrition for MCH has traditionally been considered as insufficient caloric or other nutrient intake leading to insufficient physical growth (stunting), rapid weight loss or failure to gain weight (wasting), cognitive impairment, exacerbation of anaemia and blindness, or weakening of the immune system resulting in increased risk of infectious diseases and mortality.^14^ Malnutrition for NCDs has historically been considered as excess consumption of certain nutrients such as fat, sugar, and salt as well as calories.^12^, ^15^ Yet, malnutrition for MCH and NCDs are frequently coexisting consequences of poor diet quality within populations, households, or individuals across the lifespan.^16^, ^17^ A unified global dietary quality metric (hereafter referred to as dietary metric) would aid in policy and programme decision making around improving diet in which components that contribute to both MCH and NCDs would be represented in the same assessments, and that the relative contributions of malnutrition for MCH and NCDs could be characterised and compared across settings in a standardised way.

The optimal dietary metric for assessing malnutrition for both MCH and NCDs remains uncertain. Previous reviews have described the development and characteristics of dietary metrics.^9^, ^18^, ^19^, ^20^, ^21^, ^22^, ^23^, ^24^ Some reviews qualitatively summarised the evidence for dietary metrics in relation to a single health outcome (eg, type 2 diabetes, obesity)^25^, ^26^ and others focused on several MCH^10^ or NCD outcomes.^20^, ^27^, ^28^, ^29^, ^30^ However, to our knowledge, the comprehensive reviews of dietary metrics in relation to several MCH or NCD outcomes^10^, ^20^, ^27^, ^28^, ^29^, ^30^ were done more than a decade ago, and no review has focused on both MCH and NCD outcomes together. To understand whether any existing dietary metrics are validated against MCH or NCDs, or both, and what researchers and health organisations are using to measure various aspects of the dietary contribution to MCH and NCDs, we extensively reviewed and assessed the validity of existing multinational dietary metrics for MCH and NCD health outcomes.

**Key messages**

• Many global efforts are focused on tackling the double burden of malnutrition including the UN Sustainable Development Goals. However, these targets might not be achieved without a practical and valid dietary metric to reduce malnutrition across global contexts.
• We identified 19 dietary metrics, including seven developed for maternal and child health (MCH), 12 developed for non-communicable diseases (NCDs) and none developed or applied for both.
• All MCH dietary metrics used foods only, while most NCD metrics included foods and nutrients. The most frequent components for both MCH and NCD dietary metrics were vegetables, fruits, grains, roots, and tubers.
• When the validity was assessed, we found that four NCD metrics had convincing evidence of protective associations with specific NCD outcomes, primarily mortality, cardiovascular disease, type 2 diabetes, and total cancer. The remaining eight NCD dietary metrics and all seven MCH dietary metrics did not.
• We found that few dietary metrics have been validated against MCH or NCD health outcomes, and none for both indicates. More work is needed to validate existing and novel dietary metrics for MCH and NCD, an approach that is likely critical to the achievement of global nutrition and health targets.

## Dietary metric definition and inclusion

We defined a dietary metric as a metric derived from nutrients or food or food groups, or both, with the aim of measuring dietary diversity (number of different foods consumed), or nutrient adequacy (achievement of recommended intakes of energy or essential nutrients).^10^ We included dietary metrics with the reported intended use of relating diet to MCH (micronutrient adequacy, mortality in children <5 years, maternal mortality, underweight, stunting, wasting, infectious diseases, diarrhoeal disease) and NCD health outcomes (all-cause mortality, cardiovascular disease, type 2 diabetes, gestational diabetes; total cancer and subtypes, and anthropometrics in adults and children), and that quantified the level of intake of foods or nutrients consumed or the achievement of recommended intakes. A detailed description of the health outcomes is shown in the appendix (p 1). We selected the health outcomes in accordance with WHO's categories of malnutrition.^31^ Given the interest in dietary metrics that can be used globally, we included dietary metrics that were used for this purpose in at least three countries. We did not include dietary metrics that included non-dietary factors as a core component, that only summarised a single nutrient or food, or that were developed with a primary focus on non-health outcomes (eg, sustainability). We did not include dietary metrics derived primarily from statistical data clustering approaches, such as cluster or factor analysis, because these are often not generalisable.^32^ We also did not include dietary metrics at the population level (eg, food supply) because we were interested in individual-level and household-level dietary metrics.

## Literature searches

Given the abundance of potential dietary metrics, we did not systematically identify all possible metrics. First, we compiled a list of dietary metrics through expert contacts, who were faculty members at the Friedman School of Nutrition Science, Tufts University, with expertise in food policy, agriculture, economics, international nutrition programming, humanitarian emergency relief, clinical nutrition, and epidemiology. This initial list was then used to search PubMed and Google to identify studies and development reports from government agencies, health organisations, and non-profit organisations up until Sept 21, 2018, that included these dietary metrics, met our inclusion criteria, and were published in English. These searches were complemented by hand-searching of the citations of all identified articles and reports to identify additional potential dietary metrics. We documented all identified dietary metrics that both did and did not meet the scope of our inclusion criteria (appendix p 5).

To assess validity of each relevant dietary metric, we systematically searched PubMed from Oct 11, 2000, to April 17, 2020, to identify meta-analyses or narrative reviews (including systematic reviews without a meta-analysis, but not umbrella reviews) evaluating these dietary metrics in relation to MCH and NCD health outcomes. The search terms and restrictions are described in full in the appendix (pp 2–4).

## Data extraction

VM did the literature searches, assessed titles and abstracts of all identified studies and reviewed and extracted relevant data by hand using a standardised electronic spreadsheet. The intended purpose, food and nutrient components, scoring, reference period, validity, and reliability was tabulated on each dietary metric. For each published meta-analysis or narrative review, data were extracted on the number of studies included, pooled relative risks and corresponding uncertainty, sample sizes, number of events, follow-up durations, unit of exposures, and population characteristics. In most cases, the total number of participants was not reported in the original meta-analyses and was computed from summary tables of the individual studies. Because narrative reviews do not provide pooled estimates, the extractions were done for each individual study included in the narrative review. When more than one meta-analysis or narrative review was identified for each dietary metric-health outcome relationship, we included all published meta-analyses or narrative reviews. Questions or uncertainties related to article screening and extraction were resolved by discussion with another investigator (DM).

## Assessment and grading of validity

Two investigators (VM and then either PW, RM, or DM), independently and in duplicate, used the most comprehensive (with the greatest number of participants and studies) or recent meta-analysis when available, followed by the most comprehensive (with the greatest number of participants and studies) or recent narrative review to assess and grade the evidence for validity of dietary metrics in relation to MCH and NCD health outcomes. In cases in which the most recent meta-analysis or narrative review was not the most comprehensive, we selected the most comprehensive. Each assessor extracted data on the number of studies and the direction of effects across studies (positive association, null association, negative association). If the number of studies with null associations was equal or greater to the number of studies with positive or negative associations, the relationship was classified as no association. For both meta-analyses and narrative reviews, we considered the direction of effects from each included individual study. The validity of each dietary metric was assessed using two criteria: first, the number of studies, and second, the consistency of evidence from meta-analyses and narrative reviews of prospective cohort studies or randomised controlled trials. Consistency was defined as the association is repeatedly observed in different populations and circumstances. For a consistent relationship, at least half of the associations were in the same direction and for an inconsistent relationship, fewer than half of the associations were in the same direction. Three assessment categories were established: consistent evidence for the dietary metric-health outcome relationship (positive, null, negative) from five or more studies; inconsistent evidence for the dietary metric-health outcome relationship from five or more studies; and little evidence from less than five studies. For assessing validity of each dietary metric against foods, nutrients, and other non-dietary metrics (eg, biomarkers, food insecurity indicators) we used the published papers or development reports that described the development process of the dietary metric. All disagreements among reviewing investigators regarding the assessment and grading of validity were resolved through discussion.

## Identified dietary metrics

In total, we identified 19 dietary metrics used in three or more countries each to assess diet quality in relation to various health outcomes (Table 1, Table 2, Table 3). Seven dietary metrics were primarily used for MCH: Dietary Diversity Score (DDS), Food Consumption Score (FCS), Food Variety Score (FVS), Household Dietary Diversity Score (HDDS), Infant and Young Child Minimum Dietary Diversity (IYCMDD), Minimum Dietary Diversity for Women (MDD-W), and Women's Dietary Diversity Score (WDDS) and Individual Dietary Diversity Score (IDDS). Twelve dietary metrics were most commonly used for NCDs: Alternative Healthy Eating Index (AHEI), Dietary Approaches to Stop Hypertension (DASH), Dietary Guidelines for Americans Adherence Index (DGAI), Dietary Inflammatory Index (DII), Dietary Quality Index International (DQI-I), Healthy Eating Index-2010 (HEI), Mediterranean Diet Quality Index for Children and Teenagers (KIDMED), Mediterranean Diet Score (MED), Prospective Urban Rural Epidemiology Diet Score (PURE), Recommended Foods Score (RFS), WHO Healthy Diet Metric (WHO-HDI), and World Cancer Research Fund and American Institute for Cancer Research Dietary Recommendations (WCRF-AICR). No dietary metrics were identified that were used to assess both MCH and NCDs.

**Table 1:** Dietary metrics used for assessing maternal and child health

	**Foods or nutrients and reference period**		**Scoring and cutoffs**		
	Item list	Reference period	Calculation	Range	Cutoffs or classification
DDS*^33^, ^34^					
Metric focuses on the mean number of major food groups consumed; the original DDS was developed using data from the National Health and Nutrition Examination Survey; multiple versions of the DDS have been used, including adaptations for children^35^	Dairy; meat; grain; fruit; vegetable	24 h	Count of food groups consumed	0 to 5	No cutoff
**FCS**^36^, ^37^, ^38^					
Metric focuses on predicting adequate food quantity or calorie consumption per capita in households from low-income and middle-income countries	Main staples (cereals and cereal products, roots and tubers); pulses; vegetables; fruit; meat or fish; milk; sugar; oil	7 days	Frequency-weighted score calculated using the frequency of consumption of food groups consumed by a household during reference period; data on food frequency are grouped into food groups and the consumption frequencies for each food items within a group are summed to yield a score for the food group; any food group score >7 is truncated at 7; values obtained for each food group are multiplied by weights (weights range from 0·5 to 4·0 and are based on nutrient density) to create weighted food group scores; the weights for each food group are sugar and oil (0·5), vegetables and fruit (1·0), staples (2·0), pulses (3·0), and meat or fish and milk (4·0); the weights are summed	0 to 112	Usual cutoffs are 0 to 21 (poor), 21·5 to 35·0 (borderline), and >35 (acceptable); cutoff for locations where oil and sugar are consumed daily are 0 to 28 (poor), 28·5 to 42·0 (borderline), and >42 (acceptable)
**FVS**^35^, ^39^, ^40^					
Metric focuses on the number of unique foods consumed during the reference period; commonly used among children ≤5 years as a measure of dietary diversity	Not applicable	24 h	Count of food groups consumed	Not applicable	Not specified
**HDDS**^41^, ^42^, ^43^					
Metric focuses on whether the number of unique foods consumed over a given period is a good measure of household food access in urban and rural areas; HDDS is typically measured in the person primarily responsible for food preparation in the household	Cereals; roots and tubers; legumes, nuts, and seeds; dairy; meat; fish; eggs; vegetables; fruit; oils and fats; sweets; spices, condiments, and beverages (Food and Nutrition Technical Assistance Project); and cereals; white roots and tubers; legumes, nuts, and seeds; milk and milk products; organ meat; flesh meat; fish and seafood; eggs; vitamin A-rich vegetables and tubers; dark green leafy vegetables; other vegetables; vitamin A-rich fruits; other fruits; oils and fats; sweets; spices, condiments, and beverages (Food and Agriculture Organization)	24 h	Count of food groups consumed	0 to 12	Not specified
**IYCMDD**^44^, ^45^					
Metric focuses on dietary diversity as a marker of micronutrient adequacy for ten nutrients (thiamin, riboflavin, vitamin B-6, folate, vitamin C, vitamin A, calcium, zinc, iron, and without iron separately) in children aged 6 to 23 months (both breastfed and non-breastfed) in low-income and middle-income countries; WHO recommended metric of infant and young child feeding practices	Grains, roots, and tubers; legumes and nuts; dairy; flesh foods (meat, fish, poultry and liver or organ meats); eggs; vitamin A-rich fruits and vegetables; other fruits and vegetables	24 h	Count of food groups consumed	0 to 7	WHO guidelines on infant and young child feeding practices defines minimum dietary diversity ≥4 food groups consumed
**MDD-W**^46^, ^47^					
Metric focuses on dietary diversity as a marker of micronutrient adequacy for 11 nutrients (thiamin, riboflavin, niacin, vitamin B-6, folate, vitamin B-12, vitamin C, vitamin A, calcium, iron, zinc) in women of reproductive age (15 to 49 years; both non-pregnant non-lactating and lactating women) in low-income and middle-income countries	Grains, white roots and tubers, and plantains; pulses; nuts and seeds; dairy; meat, poultry, and fish; eggs; dark green leafy vegetables; other vitamin A-rich fruits and vegetables; other vegetables; other fruits	24 h	Count of food groups consumed	0 to 10	Recommendation is ≥5 food groups consumed
**WDDS and IDDS**^42^, ^47^					
Metric focuses on the probability of micronutrient density in the diet of women of reproductive age (15 to 49 years; WDDS) and most commonly used in children aged 6–23 months (IDDS) in low-income and middle-income countries	Cereals; white roots and tubers; legumes, nuts, and seeds; dairy; organ meat; flesh meat; fish; eggs; vitamin A-rich vegetables and tubers; dark green leafy vegetables; other vegetables; vitamin A-rich fruits; other fruits; oil and fats; sweets; condiments that are aggregated into the following food groups of starchy staples; legumes, nuts, and seeds; milk and milk products; organ meat; meat and fish; eggs; dark green leafy vegetables; other vitamin A-rich fruits and vegetables; other fruits and vegetables	24 h	Count of food groups consumed	0 to 9 or 0 to 16 depending on whether further aggregation occurs	No universal cutoff; Recommendation is to use mean value or distribution to identify cutoff for the specific population

The modifications and adaptations to dietary metrics noted are not exhaustive. DDS=Dietary Diversity Score. FCS=World Food Programme's Food Consumption Score. FVS=Food Variety Score. HDDS=Household Dietary Diversity Score. IYCMDD=Infant and Young Child Minimum Dietary Diversity. MDD-W=Minimum Dietary Diversity for Women. WDDS=Women's Dietary Diversity Score. IDDS=Individual Dietary Diversity Score.

* The DDS was classified as a metric for maternal and child health because it has primarily been used for this purpose despite being originally developed for chronic diseases.

**Table 2:** Dietary metrics used for assessing non-communicable disease risk

	**Foods or nutrients and reference period**		**Scoring and cutoffs**		
	Item list	Reference period	Calculation	Range	Cutoffs or classification
**AHEI-2010**^48^					
Metric is an alternative version of the HEI that focuses on adherence to a dietary pattern associated with chronic disease risk; the AHEI was revised in 2010 to incorporate new scientific evidence on diet and health and is based on a comprehensive literature review and expert discussions to identify foods and nutrients robustly associated with low risk of chronic diseases	Vegetables; fruit; whole grains; sugar-sweetened beverages; nuts and legumes; red and processed meat; trans fat; long-chain (n-3) fats (eicosapentaenoic acid and docosahexaenoic acid); polyunsaturated fat; sodium; alcohol	Food frequency questionnaire	Components are scored from 0 (worst) to 10 (best) based on specified recommended intake for each component; the scoring for intermediate intake is not well described; recommended intake was determined a priori using the HEI recommendations, upper range of dietary guidelines (US and American Heart Association), and population distributions	0 to 110	Not specified
**DASH**^49^					
Metric developed to measure adherence to the DASH diet, a dietary pattern used in randomised controlled feeding trials to lower blood pressure in people with hypertension; multiple variations of the DASH score have been used in the literature and the DASH score described by Fung et al (2008)^50^ is the most commonly used in the literature among US populations^51^	Fruits; vegetables; nuts and legumes; low-fat dairy products; whole grains; sodium; sweetened beverages; red and processed meats (2008 version)	Food frequency questionnaire	For each component, sex-specific intake quintiles (Q) are computed, and a component score is assigned for each quintile; for fruits, vegetables, nuts and legumes, low-fat dairy products, and whole grains Q1 is assigned a value of 1, Q2 a value of 2, Q3 a value of 3, Q4 a value of 4, and Q5 a value of 5; alternatively for sodium, red and processed meats, and sweetened beverages Q1 is assigned a value of 5, Q2 a value of 4, Q3 a value of 3, Q4 a value of 2 and Q5 a value of 1; the component scores are summed (2008 version)	5 to 40	Not specified
**DGAI**^52^, ^53^, ^54^, ^55^, ^56^, ^57^, ^58^					
Metric describes adherence to the key dietary recommendations in the 2005 Dietary Guidelines for Americans except for two recommendations for special populations (eg, individuals who should not consume alcohol)	Dark green vegetable; orange vegetable; legume; starchy vegetable; other vegetable; fruit; variety of fruits and vegetables; meat and legume; milk and milk products; grain; discretionary energy (food intake subscore); whole grain; fibre; low-fat choices; total fat; saturated fat; trans-fat; cholesterol; alcohol; sodium (healthy choice subscore)	Food frequency questionnaire	A score of 1 is assigned when intake meets the recommendation, 0·5 for intake >33% of the recommendation, and 0 for intake <33% of the recommendation	0 to 20	Not specified
**DII**^59^, ^60^, ^61^					
Metric classifies an individuals' diet from pro-inflammatory to anti-inflammatory based on six inflammatory markers (IL-1β, IL-4, IL-6, IL-10, TNF-α, CRP)	Alcohol; vitamin B12; vitamin B6; β-carotene; caffeine; carbohydrate; cholesterol; energy; eugenol; total fat; fibre; folic acid; garlic; ginger; iron; magnesium; monounsaturated fat; niacin; n-3 fatty acids; n-6 fatty acids; onion; protein; polyunsaturated fat; riboflavin; saffron; saturated fat; selenium; thiamin; trans-fat; turmeric; vitamin A; vitamin C; vitamin D; vitamin E; zinc; green or black tea; flavan-3-ol; flavones; flavonones; anthocyanidins; isoflavones; pepper; thyme or oregano; rosemary	Food frequency questionnaire or 24 h	Dietary data are linked to the globally representative world database and the mean and standard deviation for each component are used as multipliers; the standard global mean is subtracted from each individual's reported amount, divided by the standard deviation and converted to a centred percentile score; the centred percentile score for each component for each individual is multiplied by the respective food parameter effect score (obtained from a literature review) to obtain a food parameter-specific score, which are summed to create an overall score; more negative scores represent anti-inflammatory diet, whereas more positive score represent pro-inflammatory diet	Approximate −10 to 10	Not specified
**DQI-I**^62^, ^63^, ^64^					
Metric was designed to promote aspects of a healthy diet in relation to major, diet-related chronic diseases and allow for international comparisons; DQI-I is a modified version of existing dietary metrics including the DQI, Institute of Nutrition and Food Hygiene-University of North Carolina at Chapel Hill Diet Quality Index, DQI-Revised, and HEI; other versions include: Med-DQI, Aussie-DQI, DQI-K, C-DQI, RC-DQI, DQI-CH	Meat, poultry, fish, or egg; dairy or beans; grains; fruits and vegetables (variety food groups); meat; poultry; fish; dairy; beans; eggs (variety protein sources); vegetables; fruit; grain; fibre; protein; iron; calcium and vitamin A (adequacy); total fat; saturated fat; cholesterol; sodium; empty calorie foods (moderation); macronutrient ratio; fatty acid ratio (overall balance)	Usual diet measured through multiple 24 h reference periods or food frequency questionnaire, or both	Variety is scored from 0 to 20, a score of 20 is allocated if at least one serving of food per day from all five food groups is consumed, if any of the food groups are not consumed, each food group consumed is scored 3 points each, maximum score of 15; adequacy is scored based on the percentage attainment of recommended intakes of eight components on a continuous scale, scoring ranges from 0 points for 0% to 5 points for 100% for each component, score range of 0 to 40; moderation is scored from 0 to 30 with a maximum of 6 points for each of the five components, intake of the components is scored as tiers with 0 points for the bottom tier, 3 points for the middle tier and 6 points for the highest tier; overall balance is scored from 0 to 10 and consists of macronutrient ratio, which is scored from 0 to 6 points based on four tiers in 2-point increments and fatty acid ratio, which is scored on three tiers in 2-point increments	0 to 100	No cutoff
**HEI-2010**^65^, ^66^, ^67^, ^68^, ^69^					
Metric describes adherence to the 2010 Dietary Guidelines for Americans; Other variations are HEI-2005 and HEI-2015 based on the corresponding year of US Dietary Guidelines, Chinese HEI, and HEI-Canada	Total fruit (includes fruit juice); whole fruit (includes all forms except juice); total vegetables; greens and beans; whole grains; refined grains; dairy; total protein foods; seafood and plant proteins; fatty acids (polyunsaturated, monounsaturated, and saturated); sodium; empty calories (energy from solid fats, alcohol, and added sugar)	Food frequency questionnaire	Maximum 5 points for total fruit, whole fruit, total vegetables, greens and beans, total protein foods, seafood and plant protein; maximum 10 points for whole grains, dairy, fatty acids, refined grains, sodium; maximum 20 points for empty calories; maximum score for each component is based on 2010 US dietary guidelines; the component scores are summed	0 to 100	Not specified
**KIDMED**^70^, ^71^, ^72^, ^73^, ^74^, ^75^, ^76^, ^77^					
Metric describes adherence to the Mediterranean diet pattern in adolescents	Components grouped into favourable and non-favourable; favourable components are daily fruit or fruit juice, eats second fruit serving daily, one daily serving of fresh or cooked vegetables, >1 daily serving of fresh or cooked vegetables, fish (2 to 3/week), legumes consumed >1/week, pasta or rice ≥5/week, cereals or grains consumed for breakfast, nuts >2 to 3/week, uses olive oil at home, dairy for breakfast (eg, yoghurt, milk), or ≥2 daily yoghurt or cheese (40 g); non-favourable components are fast food consumed >1/week, skips breakfast, commercial baked goods or pastries for breakfast, and sweets and candy several times per day	Food frequency questionnaire	Beneficial items are assigned a value of 1 when met or 0 when not met, and non-beneficial items are assigned a value of −1 when met and 0 when not met; the component scores are summed	0 to 12	Poor adherence is 0 to 3, average adherence is 4 to 7, and good adherence is 8 to 12
**MED**^78^, ^79^, ^80^, ^81^					
Metric describes adherence to the Mediterranean diet pattern in adults; variations of the MED (MDS [an alternative published abbreviation for MED], rMED, MSDPS, aMDS) exist and have been used in populations including Denmark, France, Germany, UK, Spain, Netherlands, Norway, Sweden, Italy, Switzerland, Belgium, Portugal, Hungary, Canada, USA, Japan, China and Australia^23^, ^82^, ^83^	Fruits, vegetables, legumes, cereals, meat and meat products, dairy, monounsaturated fatty acids-saturated fatty acids (MUFA-SFA) ratio, and alcohol (1999 version); fruits and nuts, vegetables, legumes, cereals, meat and meat products, dairy, MUFA-SFA ratio, alcohol, and fish (2003 version)	Food frequency questionnaire	Scoring not described; for 1999 version; for the 2003 version calculate sex-specific medians; intake below the median for beneficial components (vegetables, legumes, fruits and nuts, cereal, and fish) are assigned a value of 0, and intake above or at the median is assigned a value of 1; components assumed to be detrimental (meat, poultry, and dairy products) intake below the median is assigned a value of 1 and at or above the median a value of 0; for ethanol, a value of 1 is assigned to men who consume between 10 and 50 g per day and to women who consume between 5 and 25 g per day; for MUFA-SFA ratio intake at or above the median is assigned a value of 1 and 0 for below the median	0 to 9	Not specified
**PURE**^84^					
Metric focuses on the specific food groups found to be beneficially associated with the risk of mortality in a multinational prospective cohort study	Fruits; vegetables; legumes; nuts; dairy; unprocessed red meat; fish	Food frequency questionnaire	For each component, intake quintiles are computed, and a component score is assigned for each quintile; Q1 is assigned a value of 1, Q2 a value of 2, Q3 a value of 3, Q4 a value of 4, and Q5 a value of 5; the component scores are summed	7 to 35	Not specified
**RFS**^85^, ^86^, ^87^, ^88^, ^89^					
Metric measures diet quality as the consumption of foods recommended by several US dietary guidelines (US National Research Council, Surgeon General and US Department of Agriculture and Health and Human Services)	Apples, pears; oranges; cantaloupe; orange juice, grapefruit juice; grapefruit; other fruit juices; dried beans; tomatoes; broccoli; spinach; mustard, turnip, collard greens; carrots, mixed vegetables with carrots; green salad; sweet potatoes, yams; other potatoes; baked or stewed chicken or turkey; baked or broiled fish; dark breads (eg, whole wheat, rye, pumpernickel); cornbread, tortillas, grits; high-fibre cereals (eg, bran, granola, shredded wheat); cooked cereals; 2% milk and beverages with 2% milk; 1% or skim milk	Food frequency questionnaire	For each component, 1 point is allocated if consumed at least once per week; the component scores are summed	0 to 23	Not specified
**WHO-HDI**^90^, ^91^, ^92^, ^93^, ^94^					
Metric describes adherence to the WHO dietary guidelines (initially 1990 guidelines and revised to the 2003 guidelines) in European populations	Saturated fatty acids; polyunsaturated fatty acids; protein; complex carbohydrates; dietary fibre; fruits and vegetables; pulses, nuts, seeds; monosaccharides and disaccharides; cholesterol (WHO 1990 guidelines); saturated fatty acids; monosaccharides and disaccharides, cholesterol; protein; total dietary fibre; fruits and vegetables; n3-polyunsaturated fatty acids; n6-polyunsaturated fatty acids; trans fatty acids; sodium (WHO 2003 guidelines); saturated fatty acids; free sugar; total fat; total dietary fibre; fruits and vegetables; polyunsaturated fatty acids; potassium (WHO 2015 guidelines)	Food frequency questionnaire	For each component, a value of 1 is assigned if intake is in the recommended range and a value of 0 if not in the recommended range; the components are summed	0 to 7 for the WHO 2015 version	Not specified
**WCRF-AICR**^95^, ^96^, ^97^					
Metric describes adherence to the WCRF-AICR dietary recommendations	Limit consumption of energy-dense foods and sugary drinks; eat mostly foods of plant origin; limit red meat intake and avoid processed meat; limit alcoholic drinks; recommendation to limit consumption of salt and avoid mouldy cereals (grains) or pulses (legumes) was not included	Food frequency questionnaire	Each component is scored with 1 point for complete adherence, 0·5 for moderate adherence, and 0 for non-adherence for each recommendation specific cutoff; the component scores are summed	0 to 4	Not specified

The modifications and adaptations to dietary metrics noted are not exhaustive. AHEI=Alternative Healthy Eating Index. aMDS=Alternative Mediterranean Diet Score. Aussie-DQI=Australian Diet Quality Index. C-DQI=Children's Diet Quality Index. DASH=Dietary Approaches to Stop Hypertension. DGAI=Dietary Guidelines for Americans Adherence Index. DII=Dietary Inflammatory Index. DQI-CH=Dietary Quality Index for China. DQI-I=Diet Quality Index-International. DQI-K=Diet Quality Index for Koreans. HEI=Healthy Eating Index. KIDMED=Mediterranean Diet Quality Index for Children and Teenagers. MDS=Mediterranean Diet Score. MED=Mediterranean Diet Score. Med-DQI=Mediterranean Diet Quality Index. MSDPS=Mediterranean-Style Dietary Pattern Score. PURE=Prospective Urban Rural Epidemiology Diet Score. RC-DQI=Revised Children's Diet Quality Index. RFS=Recommended Foods Score. rMED=Revised Mediterranean Diet Score. WHO-HDI=WHO Healthy Diet Indicator. WCRF-AICR=World Cancer Research Fund and American Institute for Cancer Research.

**Table 3:** Estimates of aetiologic effects of dietary metrics and risk of health outcomes

	**Meta-analysis search date**	**Studies included**	**Source**	**Number of participants**	**Countries**	Unit of exposure*	**Relative risk (95% CI)**	**I^2^**†	**p value for heterogeneity**
**AHEI**									
All-cause mortality	May 15, 2017	7	Schwingshackl et al (2018)^98^	975 639	USA, China, UK	High *vs* low	0·76 (0·74 to 0·79)	71%	0·003
All-cause mortality among cancer survivors	May 15, 2017	3	Schwingshackl et al (2018)^98^	9508	USA	High *vs* low	0·85 (0·70 to 1·03)	65%	0·03
Cardiovascular disease	May 15, 2017	13	Schwingshackl et al (2018)^98^	1 296 276	USA, China, UK	High *vs* low	0·75 (0·72 to 0·77)	39%	0·05
Cardiovascular mortality	Dec 14, 2015	7	Onvani et al (2017)^99^	820 778	USA, China, UK	High *vs* low	0·74 (0·71 to 0·78)	NR	NR
Type 2 diabetes	May 15, 2017	9	Schwingshackl et al (2018)^98^	605 077	USA, Denmark, France, Germany, Italy, Spain, Sweden, UK, Netherlands	High *vs* low	0·80 (0·74 to 0·86)	76%	<0·001
Cancer	May 15, 2017	18	Schwingshackl et al (2018)^98^	3 013 168	USA, Great Britain, China, Australia	High *vs* low	0·88 (0·85 to 0·91)	54%	0·001
Cancer mortality	June 2017	9	Milajerdi et al (2018)^100^	964 740	USA, England	High *vs* low	0·90 (0·85 to 0·95)	62%	0·003
Cancer mortality among cancer survivors	May 15, 2017	3	Schwingshackl et al (2018) ^98^	9508	USA	High *vs* low	0·95 (0·79 to 1·13)	20%	0·29
**DASH**									
All-cause mortality	May 15, 2017	8	Schwingshackl et al (2018)^98^	1 353 039	USA, China, Denmark, France, Germany, Greece, Italy, Netherlands, Spain, Sweden, Norway, UK	High *vs* low	0·80 (0·79 to 0·82)	9%	0·36
All-cause mortality among cancer survivors	May 15, 2017	3	Schwingshackl et al (2018)^98^	9508	USA	High *vs* low	0·94 (0·82 to 1·08)	27%	0·25
Cardiovascular disease	May 15, 2017	18	Schwingshackl et al (2018)^98^	1 745 815	USA, Taiwan, China, UK, Denmark, France, Germany, Greece, Italy, Netherlands, Spain, Sweden and Norway	High *vs* low	0·80 (0·77 to 0·84)	49%	0·006
Coronary heart disease	January 2012	3	Salehi-Abargouei et al (2013)^101^	144 337	USA	High *vs* low	0·79 (0·71 to 0·88)	0%	0·583
Coronary artery disease	June 2019	7	Yang et al (2019)^102^	377 725	USA, UK, Netherlands	High *vs* low	0·82 (0·78 to 0·87)	0%	0·53
Total stroke	May 2018	11	Feng et al (2018)^103^	474 228	USA, Hong Kong, Taiwan, Italy, Sweden, Germany, UK, Netherlands	High *vs* low	0·88 (0·83 to 0·93)	4%	NR
Type 2 diabetes	May 15, 2017	8	Schwingshackl et al (2018)^98^	258 893	USA, Denmark, France, Germany, Italy, Spain, Sweden, UK, Netherlands	High *vs* low	0·80 (0·74 to 0·86)	61%	0·01
Cancer	May 15, 2017	14	Schwingshackl et al (2018)^98^	2 987 645	USA, Sweden, China, Denmark, France, German, Greece, Italy, Netherlands, Spain, Norway, UK	High *vs* low	0·82 (0·80 to 0·86)	48%	0·007
Cancer mortality	July 2018	9	Ali Mohsenpour et al (2019)^104^	1 414 944	USA, China, Denmark, France, Germany, Greece, Italy, Netherlands, Norway, Spain, UK, Sweden, Singapore	High *vs* low	0·84 (0·81 to 0·86)	13%	0·323
Cancer mortality among cancer survivors	May 15, 2017	3	Schwingshackl et al (2018)^98^	9508	USA	High *vs* low	0·93 (0·79 to 1·10)	0%	0·73
Colorectal cancer	April 2019	6	Mohseni et al (2020)^105^	836 218	USA, Canada	High *vs* low	0·81 (0·75 to 0·88)	54%	0·017
Colon cancer	July 2018	2	Ali Mohsenpour et al (2019)^104^	624 587	USA	High *vs* low	0·80 (0·74 to 0·87)	0%	0·922
Rectal cancer	July 2018	2	Ali Mohsenpour et al (2019)^104^	624 287	USA	High *vs* low	0·84 (0·74 to 0·96)	16%	0·274
Weight loss in adults, kg	December 2015	10	Soltani et al (2016)^106^	1291	USA, Australia, Iran	DASH diet *vs* control diet	−1·42 (−2·03 to −0·82)	71%	<0·001
Body-mass index in adults, kg/m^2^	December 2015	6	Soltani et al (2016)^106^	1157	USA, Iran and China	DASH diet *vs* control diet	−0·42 (−0·64 to −0·20)	82%	0·01
Waist circumference in adults, cm	December 2015	2	Soltani et al (2016)^106^	511	USA, Iran	DASH diet *vs* control diet	−1·05 (−1·61 to −0·49)	80%	<0·001
**DDS**									
Cancer mortality	June 2017	2	Milajerdi et al (2018)^100^	12 080	USA, Taiwan	High *vs* low	1·03 (0·59 to 1·82)	63%	0·068
**DII**									
All-cause mortality	NR	5	Shivappa et al (2017)^59^	99 147	UK, USA, Sweden, France	High *vs* low	1·04 (1·03 to 1·05)	53%	0·074
Cardiovascular mortality	NR	4	Shivappa et al (2017)^59^	91 260	UK, USA, Sweden	High *vs* low	1·05 (1·03 to 1·07)	15%	0·319
Cancer mortality	NR	5	Shivappa et al (2017)^59^	99 142	UK, USA, Sweden, France	High *vs* low	1·05 (1·03 to 1·07)	30%	0·22
Breast cancer	February 2017	5	Zahedi et al (2018)^107^	279 402	USA, Sweden, France	High *vs* low	1·04 (0·98 to 1·10)	31%	0·218
Gastric cancer‡	December 2018	3	Du et al (2019)^108^	2118	Italy, Korea, Iran	Low *vs* high	2·11 (1·41 to 3·15)	41%	0·19
**DQI**									
Cancer mortality	June 2017	5	Milajerdi et al (2018)^100^	599 041	Sweden, USA, Spain, England	High *vs* low	0·91 (0·89 to 0·93)	2%	0·420
**HEI**									
All-cause mortality	May 15, 2017	8	Schwingshackl et al (2018)^98^	1 328 413	USA, Denmark, France, Germany, Greece, Italy, Netherlands, Norway, Spain, Sweden, UK	High *vs* low	0·78 (0·76 to 0·80)	37%	0·11
All-cause mortality among cancer survivors	May 15, 2017	5	Schwingshackl et al (2018) ^98^	12 040	USA	High *vs* low	0·85 (0·75 to 0·96)	26%	0·24
Cardiovascular disease	May 15, 2017	11	Schwingshackl et al (2018) ^98^	1 600 121	USA, Denmark, France, Germany, Greece, Italy, Netherlands, Norway, Spain, Sweden, UK	High *vs* low	0·79 (0·77 to 0·82)	16%	0·28
Cardiovascular mortality	Dec 14, 2015	5	Onvani et al (2017)^99^	740 455	USA	High *vs* low	0·79 (0·76 to 0·83)	NR	NR
Type 2 diabetes	May 15, 2017	3	Schwingshackl et al (2018)^98^	303 213	USA	High *vs* low	0·87 (0·82 to 0·93)	61%	0·05
Cancer	May 15, 2017	21	Schwingshackl et al (2018)^98^	5 048 954	USA, Denmark, France, Germany, Greece, Italy, Netherlands, Norway, Spain, Sweden, UK	High *vs* low	0·83 (0·79 to 0·87)	73%	<0·001
Cancer mortality	Dec 14, 2015	6	Onvani et al (2017)^99^	741 091	USA, China	High *vs* low	0·80 (0·76 to 0·83)	NR	NR
Cancer mortality among cancer survivors	May 15, 2017	5	Schwingshackl et al (2018)^98^	12 040	USA	High *vs* low	0·84 (0·73 to 0·97)	18%	0·30
**IYCMDD**									
Stunting‡	November 2017	5	Berhe et al (2019)^109^	NR	Ethiopia	<4 score *vs* ≥4 score	1·95 (1·31 to 2·92)	72%	0·006
**MED**									
All-cause mortality	NR	7	Bonaccio et al (2018)^110^	11 738	Australia, Greece, Sweden, UK, Italy, Belgium, Denmark, France, Netherlands, Portugal, Spain, Switzerland	1-point increase	0·95 (0·93 to 0·96)	0%	0·47
Cardiovascular disease	August 2016	11	Rosato et al (2017)^111^	758 280	USA, Spain, Sweden, UK, Netherlands, Italy, Finland	High *vs* low	0·81 (0·74 to 0·88)	80%	<0·0001
Cardiovascular mortality	May 7, 2018	21	Becerra-Tomas et al (2019)^112^	883 878	USA, UK, Denmark, Spain, Switzerland, Italy, Australia, Sweden	High *vs* low	0·79 (0·77 to 0·82)	0%	0·64
Coronary heart disease	August 2016	11	Rosato et al (2017)^111^	379 473	USA, Spain, Sweden, Netherlands, Greece, Italy, Finland	High *vs* low	0·70 (0·62 to 0·80)	45%	0·06
Coronary heart disease mortality	May 7, 2018	6	Becerra-Tomas et al (2019)^112^	270 565	USA, UK, Australia, Sweden	High *vs* low	0·73 (0·59 to 0·89)	63%	0·02
Myocardial infarction	June 2014	3	Grosso et al (2017)^113^	44 428	USA, Germany, Sweden	High *vs* low	0·67 (0·54 to 0·83)	NR	NR
Total stroke§	August 2016	6	Rosato et al (2017)^111^	181 353	USA, China, Netherlands, Greece, Italy, Australia	High *vs* low	0·73 (0·59 to 0·91)	46%	0·10
Ischaemic stroke	August 2016	5	Rosato et al (2017)^111^	206 562	USA, Sweden, Italy	High *vs* low	0·82 (0·73 to 0·92)	0%	0·46
Haemorrhagic stroke	August 2016	4	Rosato et al (2017)^111^	203 994	USA, Sweden, Italy	High *vs* low	1·01 (0·74 to 1·27)	36%	0·20
Stroke mortality	May 7, 2018	4	Becerra-Tomas et al (2019)^112^	195 644	Greece, USA, UK, Denmark, Sweden	High *vs* low	0·87 (0·80 to 0·96)	0%	0·74
Type 2 diabetes	Dec 31, 2015	6	Jannasch et al (2017)^25^	196 772	USA, Spain, Greece, Denmark, France, Germany, Italy, Sweden, UK, Netherlands	High *vs* low	0·87 (0·82 to 0·93)	26%	0·24
Cancer mortality	June 2017	6	Milajerdi et al (2018)^100^	789 104	USA	High *vs* low	0·81 (0·78 to 0·83)	2%	0·420
Breast cancer	August 2016	5	Van den Brandt et al (2017)^114^	58 923	USA, UK, Sweden, Netherlands, Denmark, France, Germany, Greece, Italy, Norway, Spain	High *vs* low	0·94 (0·88 to 1·01)	13%	0·33
Gastric cancer	December 2018	2	Du et al (2019)^108^	956 518	USA, Denmark, UK, France, Sweden, Germany, Italy, Spain, Netherlands, Norway, Greece	High *vs* low	0·89 (0·68 to 1·17)	52%	0·10
Weight loss in adults, kg	June 2010	12	Esposito et al (2011)^115^	2683	Italy, USA, France, Israel, Greece, Spain, Germany, Netherlands	MED diet *vs* control diet	−1·75 (−2·86 to −0·64)	95%	0·001
Body-mass index in adults, kg/m^2^	June 2010	15	Esposito et al (2011)^115^	3337	Italy, USA, France, Israel, Greece, Spain, Germany	MED diet *vs* control diet	−0·57 (−0·93 to −0·21)	92%	<0·001
Waist circumference in adults, cm	Feb 9, 2016	29	Garcia et al (2016)^116^	4133	Canada, Algeria, Netherlands, UK, Spain, Italy, USA, Greece, Chile, Sweden, Australia, Romania, South Africa	MED *vs* control diet	−0·44 (−0·48 to −0·41)	96%	<0·0001

Summary of the meta-analyses finding used for grading the evidence for associations. AHEI=Alternative Healthy Eating Index. DASH=Dietary Approaches to Stop Hypertension. DDS=Dietary Diversity Score. DII=Dietary Inflammatory Index. DQI-I=Diet Quality Index-International. HEI=Healthy Eating Index. IYCMDD=Infant and Young Child Minimum Dietary Diversity. MED=Mediterranean Diet Score. NR=not reported.

* High versus low dietary metrics (categorical), point increase in score (continuous), or trial experimental and control groups.

† Values are rounded to the nearest whole number.

‡ Odds ratio and 95% CI reported.

§ Unspecified stroke considered total stroke.

## Intended purpose of the dietary metric

Of the seven MCH dietary metrics, two were assessed at the household-level (FCS, HDDS) and five at the individual level (DDS, FVS, IYCMDD, MDD-W, and WDDS and IDDS). All 12 NCD dietary metrics were assessed at the individual level. Most of the MCH dietary metrics were developed for describing micronutrient or caloric adequacy (FCS, IYCMDD, MDD-W, and WDDS and IDDS). Metrics measuring dietary diversity (DDS, FVS) or household food access (HDDS) were less common. NCD dietary metrics were designed to describe adherence to US (DGAI, HEI, RFS) or international (WHO-HDI, WCRF-AICR) dietary guidelines, diet patterns (AHEI, DASH, KIDMED, MED), or foods associated with chronic disease risk (DQI-I, PURE). One NCD dietary metric was designed to measure inflammatory potential (DII).

## Included food and nutrient components

**Figure 1:**
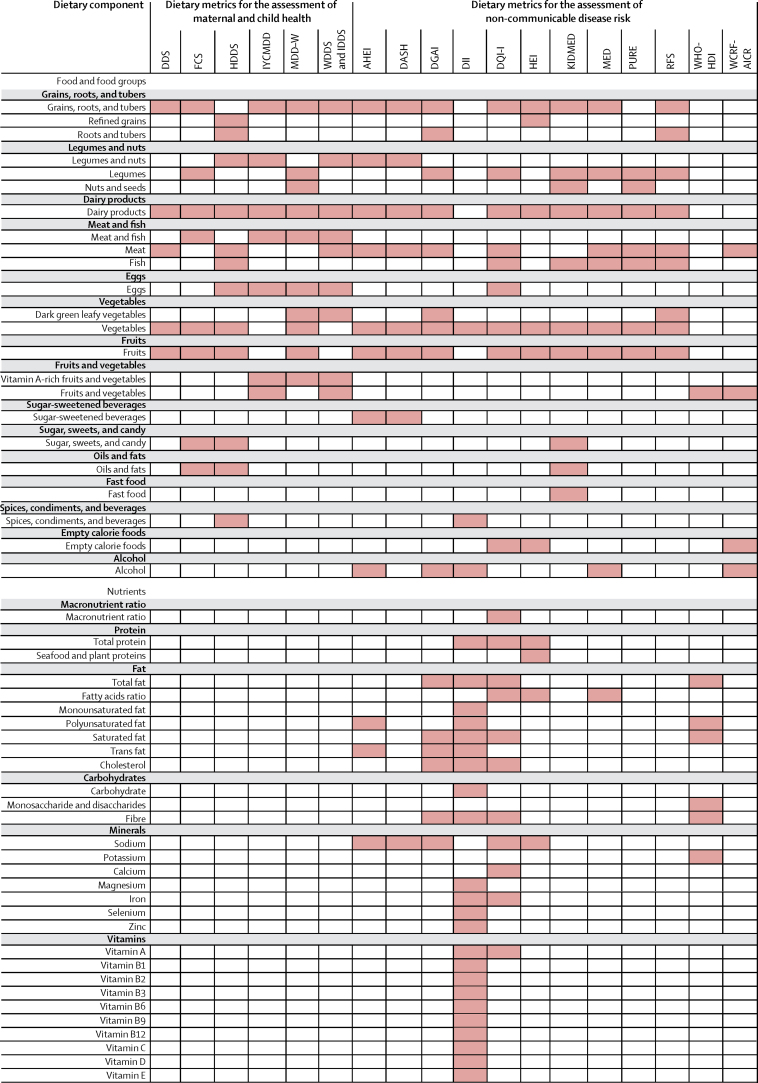
Foods and nutrients included in the dietary metrics Shading indicates that the food or nutrient is included in the dietary metric (appendix pp 22–23). DDS=Dietary Diversity Score. FCS=World Food Programme's Food Consumption Score. FVS=Food Variety Score. HDDS=Household Dietary Diversity Score. IYCMDD=Infant and Young Child Minimum Dietary Diversity. MDD-W=Minimum Dietary Diversity for Women. WDDS=Women's Dietary Diversity Score. IDDS=Individual Dietary Diversity Score. AHEI=Alternative Healthy Eating Index. DASH=Dietary Approaches to Stop Hypertension. DGAI=Dietary Guidelines for Americans Adherence Index. DII=Dietary Inflammatory Index. DQI-I=Diet Quality Index-International. HEI=Healthy Eating Index. KIDMED=Mediterranean Diet Quality Index for Children and Teenagers. MED=Mediterranean Diet Score. PURE=Prospective Urban Rural Epidemiology Diet Score. RFS=Recommended Foods Score. WHO-HDI=WHO Healthy Diet Indicator. WCRF-AICR World Cancer Research Fund and American Institute for Cancer Research.

The foods and nutrients included in each dietary metric is shown in figure 1. The median number of included food groups or nutrients per dietary metric is 9·5 (IQR 7·25–12·0). The MCH dietary metrics (8·5, IQR 7·25–9·75) included fewer food groups or nutrients compared with the NCD dietary metrics (11·5, IQR 7·75–20·25). All MCH dietary metrics (excluding the FVS which counts the number of unique foods eaten without specifying food groups or nutrients) used only food groups. Alternatively, six NCD dietary metrics used food groups only, and six used foods and nutrients. None of the identified dietary metrics used only nutrients.

Among all dietary metrics (excluding the FVS), the most frequent foods or nutrients included were vegetables (18 metrics); fruits (17 metrics); dairy products (15 metrics); and grains, roots, and tubers (14 metrics). Among MCH dietary metrics, grains, roots, and tubers, fruits, vegetables, dairy products, and meat and fish were included in all dietary metrics; other frequent foods were legumes and nuts (five metrics), and eggs (four metrics). The most common foods in NCD dietary metrics were vegetables (12 metrics) and fruits (11 metrics), followed by dairy products (nine metrics), grains, roots, and tubers (eight metrics), legumes and nuts (eight metrics), and meat (eight metrics). Uncommon food groups included spices, condiments, and beverages; fast foods; and sugar-sweetened beverages. Unhealthy food groups (eg, sugar-sweetened beverages, sugar, sweets, candy, fast food, or empty calorie foods) were included in two MCH dietary metrics (FCS, HDDS) and six NCD dietary metrics (DASH, AHEI, DQI, HEI, KIDMED, WCRF-AICR). Of the eight NCD dietary metrics that included nutrients (AHEI, DASH, DGAI, DII, DQI-I, HEI, MED, WHO-HDI), the most frequent nutrients were sodium (six of eight metrics), total fat, saturated fat, and fibre (four of eight each).

## Reference period, scoring, and cutoffs

All dietary metrics were intended to assess habitual diet, five MCH dietary metrics asked about intake in the past 24 hours, and all NCD dietary metrics asked about diet over an extended period, often the past year.

Methods of scoring varied considerably. Five MCH dietary metrics (FVS, HDDS, IYCMDD, MDD-W, and WDDS and IDDS) and four NCD dietary metrics (DDS, KIDMED, RFS, WHO-HDI) assigned a binary value if the foods or nutrients were consumed during the reference period or recommended intakes were met. Several other NCD dietary metrics (AHEI, DGAI, WCRF-AICR) scored with more than two categories. To identify scoring thresholds, three NCD dietary metrics (MED, DASH, PURE) used the intake distributions in the population in which the metric was being used (ie, using medians and quintiles). One MCH (FCS) and three NCD dietary metrics (DII, DQI-I, HEI) used complex, component-specific scoring.

Positive value only scores were used by all MCH dietary metrics and most NCD dietary metrics, except the KIDMED and DII. Four NCD dietary metrics (DASH, KIDMED, MED, PURE) assigned scores based on the perceived directional benefit of the foods or nutrients, or both, with both lower intake of unhealthy items and greater intake of healthy items receiving higher scores. Unequal scoring weights were used by one MCH dietary metric (FCS) and three NCD dietary metrics (DQI-I, DII, HEI).

Once the overall dietary metric score was determined, assessment of the score varied. Most dietary metrics (15 of 19) used continuous assessments (ie, higher is better). Two MCH dietary metrics (ICYMDD, MDD-W) specified binary cutoffs, while one MCH (FCS) and one NCD dietary metric (KIDMED) provided ordinal (multi-category) cutoffs.

## Dietary metric reliability and validity

Reliability, defined as repeated measure validity, was assessed for only two NCD dietary metrics (appendix pp 6–13). The HEI metric showed reasonable reliability for all components except sodium and dairy. The KIDMED metric showed moderate to excellent test-retest reliability.

From their development reports, three MCH dietary metrics were validated against foods or nutrients (IYCMDD, MDD-W, and WDDS and IDDS) and two MCH metrics against other non-dietary metrics (FCS, HDDS; appendix pp 6–13). Three NCD dietary metrics were validated against foods or nutrients (HEI, DGAI) or biomarkers (DII).

**Figure 2:**
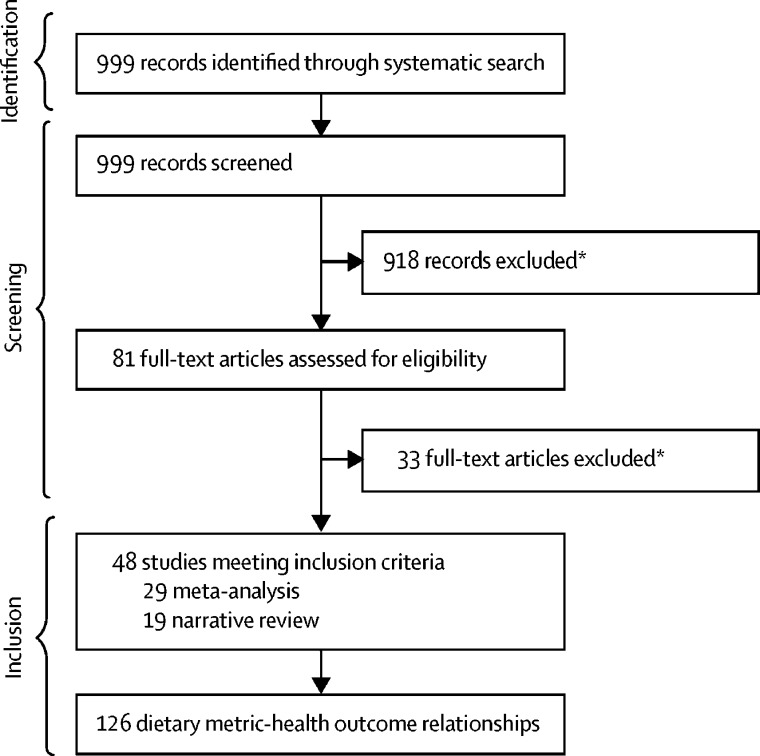
Screening and selection process of meta-analyses evaluating dietary metric-disease relationships *Study design or not relevant outcome or exposure.

**Figure 3:**
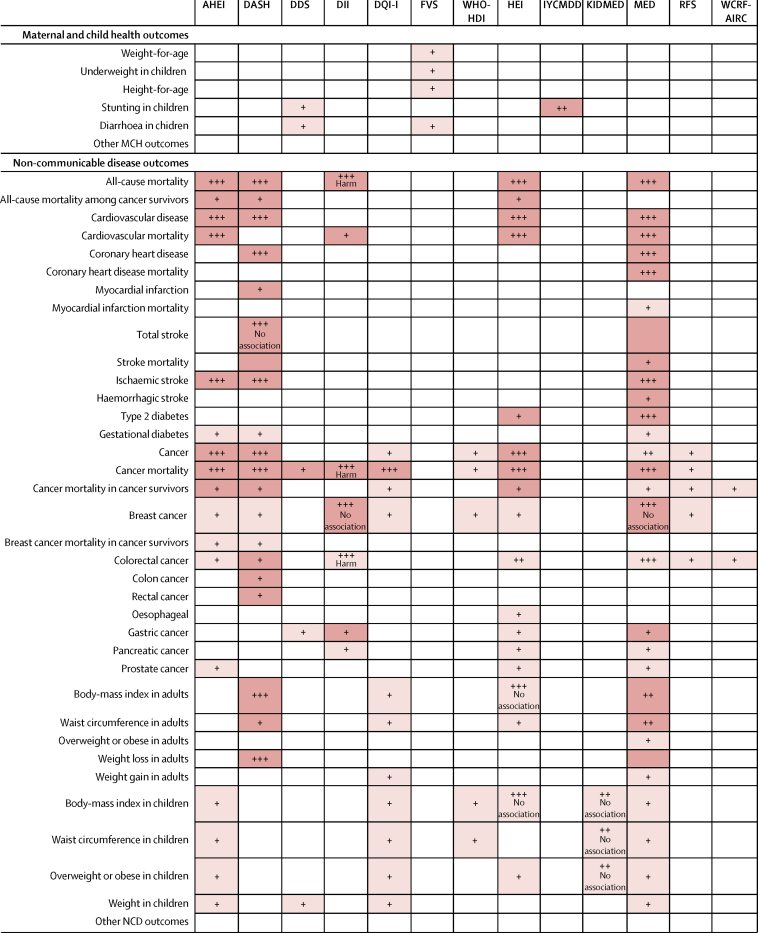
Grading of evidence for associations of dietary metrics with maternal and child health (MCH) and non-communicable diseases (NCDs) based on meta-analyses and narrative reviews Dark shading indicates a meta-analysis, light shading indicates a narrative review, and no shading indicates that no review was identified. One plus sign indicates little evidence from few studies (<5), two plus signs indicate inconsistent results from a moderate number of studies (≥5), and three plus signs indicate consistent evidence from multiple high-quality studies (≥5). The relationship between a higher dietary metric and the health outcome was protective, unless stated otherwise. AHEI=Alternative Healthy Eating Index. DASH=Dietary Approaches to Stop Hypertension. DDS=Dietary Diversity Score. DII=Dietary Inflammatory Index. DQI-I=Diet Quality Index-International. FVS=Food Variety Score. WHO-HDI=WHO Healthy Diet Indicator. HEI=Healthy Eating Index. IYCMDD=Infant and Young Child Minimum Dietary Diversity. KIDMED=Mediterranean Diet Quality Index for Children and Teenagers. MED=Mediterranean Diet Score. RFS=Recommended Foods Score. WCRF-AICR World Cancer Research Fund and American Institute for Cancer Research.

Our systematic search identified 48 meta-analyses or narrative reviews^25^, ^26^, ^59^, ^98^, ^99^, ^100^, ^101^, ^102^, ^103^, ^104^, ^105^, ^106^, ^107^, ^108^, ^109^, ^110^, ^111^, ^112^, ^113^, ^114^, ^115^, ^116^, ^117^, ^118^, ^119^, ^120^, ^121^, ^122^, ^123^, ^124^, ^125^, ^126^, ^127^, ^128^, ^129^, ^130^, ^131^, ^132^, ^133^, ^134^, ^135^, ^136^, ^137^, ^138^, ^139^, ^140^, ^141^ that assessed validity of these dietary metrics against health outcomes (figure 2). These reports included 126 dietary metric-health outcome relationships for 13 of the 19 dietary metrics. Nearly all dietary metric-health outcome relationships (116 of 126) were for NCD dietary metrics, with only ten for MCH dietary metrics. Table 3 describes the identified associations of each dietary metric and MCH and NCD health outcome relationship from published meta-analyses, and the evidence for associations based on both meta-analyses and narrative reviews is shown in figure 3.

The most commonly studied NCD dietary metrics were the MED (29 dietary metric-relationships), DASH (19 dietary metric-relationships), HEI (18 dietary metric-relationships), and AHEI (17 dietary metric-relationships). The most frequently studied outcomes were cancer (53 relationships), anthropometrics in children (21 relationships), cardiovascular disease (18 relationships), and anthropometrics in adults (13 relationships). Among our outcomes of interest, we did not find meta-analyses or narrative reviews of any of the dietary metrics in relation to major MCH outcomes including micronutrient adequacy, mortality for children younger than 5 years, maternal mortality, wasting, or infectious diseases. We also did not find meta-analyses or narrative reviews evaluating any dietary metric against both MCH and NCD outcomes.

## Evidence synthesis and grading for dietary metrics

Four of the MCH dietary metrics (FCS, HHDS, MDD-W, and WDDS and IDDS) did not have any identified meta-analyses or narrative reviews assessing their relationship with MCH or NCD health outcomes (figure 3). We found little evidence from few studies for the DDS and stunting and diarrhoea, the IYCMDD and stunting, and the FVS and child underweight.

Four of the NCD dietary metrics had consistent evidence for associations with NCD health outcomes (figure 3). The MED was found to be inversely associated with all-cause mortality, cardiovascular disease, cardiovascular disease mortality, coronary health disease, total and ischaemic stroke, type 2 diabetes, and cancer mortality. Both AHEI and HEI were associated with lower risk of all-cause mortality, cardiovascular disease, cardiovascular disease mortality, cancer, and cancer mortality, while DASH was inversely associated with all-cause mortality, cardiovascular disease, type 2 diabetes, cancer, cancer mortality, body-mass index (BMI), and weight loss in adults.

We found inconsistent evidence for MED and cancer, BMI, waist circumference, and weight loss in adults; for KIDMED and BMI, waist circumference and overweight or obesity in children; and for HEI and colorectal cancer. Six NCD dietary metrics (AHEI, DASH, DQI-I, WHO-HDI, HEI, RFS) had little evidence for associations with breast cancer. For two NCD dietary metrics (DGAI, PURE), no meta-analyses or narrative reviews were identified of relationships with MCH or NCD health outcomes.

Several dietary metrics had evidence (consistent or inconsistent) showing no association with NCD health outcomes including: DASH with total stroke; DII with breast cancer; HEI with BMI in adults and children; KIDMED with BMI, waist circumference, and overweight or obesity in children; and MED with breast cancer (figure 3).

When we looked at what characteristics of the dietary metrics might be more predictive of validity of health outcomes we found that the four NCD metrics (AHEI, DASH, HEI, MED) with consistent evidence of associations were developed to describe adherence to dietary guidelines or diet patterns, consisted of food and nutrient components, all included healthy plant foods (fruits, vegetables, whole grains, nuts and legumes) and dairy, and most included red and processed meat, or sodium.

## Discussion

In this comprehensive review of dietary metrics for assessing diet quality globally, we identified 19 dietary metrics, including seven for MCH and 12 for NCD outcomes; and none developed or applied for both. The dietary metrics varied substantially in their composition and scoring, with MCH dietary metrics generally focused on a few key foods (grains, fruits, vegetables, dairy products, meat, and fish) and NCD dietary metrics incorporating a more diverse mix of foods and nutrients, or both. Importantly, most of these dietary metrics were not validated against health outcomes in meta-analyses or narrative reviews. Only four NCD dietary metrics (MED, AHEI, HEI, DASH) had convincing evidence of protective associations, mainly for all-cause mortality, cardiovascular diseases, type 2 diabetes, total cancer, and cancer mortality. These four dietary metrics had little evidence of associations for anthropometrics in adults or children, and were not validated against MCH outcomes (micronutrient adequacy, mortality in children younger than 5 years, stunting, wasting, and infectious and diarrhoeal disease) in published meta-analyses or narrative reviews. The remaining eight NCD dietary metrics and all seven MCH dietary metrics were either not convincingly validated against most MCH and NCD health outcomes or published meta-analyses and narrative reviews assessing their validity were not identified. Additionally, our findings show that no dietary metrics currently exist designed or validated to characterise the double burden of malnutrition. To our knowledge, these findings provide the most current and extensive synthesis of specific multinational dietary metrics for risk of MCH and NCDs.

Among MCH dietary metrics, the IYCMDD is the most widely used and is routinely collected in studies in low-income and middle-income countries, such as the Demographic and Health Surveys^142^ and UNICEF Multiple Indicator Cluster Surveys,^143^ as a component of the WHO's Minimum Acceptable Diet for children aged 6–23 months.^144^, ^145^, ^146^ Despite its frequent use, we identified one meta-analysis assessing the association of the IYCMDD with one MCH health outcome, and no meta-analyses or narrative reviews with NCD outcomes. We are aware of selected individual studies that have reported beneficial associations of IYCMDD with child growth outcomes (eg, stunting, wasting, height-for-age Z score, and weight-for-height Z score).^147^, ^148^, ^149^, ^150^, ^151^, ^152^, ^153^, ^154^, ^155^ However, such evidence has not been systematically reviewed nor summarised. The IYCMDD assesses binary recall of seven broad food groups over the previous 24 hours. Although data collection is quick and undemanding for research staff and participants, the simplicity of the IYCMDD is also a potential limiting factor in predicting health outcomes. Ironically, the existing widespread use of the IYCMDD could impede development and validation of new metrics for MCH or the double burden of malnutrition, because a substantial proportion of available dietary data in low-income and middle-income countries is restricted to IYCMDD's broad food groups. Our findings show that much work is needed to validate the IYCMDD for uses other than its intended purpose of measuring micronutrient adequacy. In the absence of robust validation of the IYCMDD against health outcomes, studies and organisations should consider more rigorously validated questions on diet quality.

Among the other MCH dietary metrics, we identified only two narrative reviews for the DDS,^100^, ^118^ and four for the FVS.^118^, ^123^, ^131^, ^141^ No meta-analyses or narrative reviews were identified for the remaining MCH dietary metrics (FCS, HDDS, MDD-W, and WDDS and IDDS). We expect that individual studies will have assessed the relationship between these dietary metrics and MCH outcomes, and our findings highlight the need to pool and synthesise available individual studies of these dietary metrics in relation to both MCH and NCD outcomes in adults and children.

Our investigation also found that although numerous dietary metrics are used to assess the dietary risks of NCDs, most do not have convincing evidence of associations that have been summarised in published narrative reviews or meta-analyses. Only four metrics (MED, AHEI, HEI, DASH) had generally consistent evidence of associations with specific NCD outcomes. For outcomes such as anthropometrics and certain cancer subtypes, further validation is needed. Additionally, none of these four NCD dietary metrics have been assessed in meta-analyses or systematic reviews of important MCH outcomes.

Previous reviews of dietary metrics have focused solely on describing dietary metric features and dietary metric development,^9^, ^19^, ^20^, ^21^, ^22^, ^23^, ^24^ or the evidence for dietary metrics in relation to a single disease.^25^, ^26^ Other reviews looked more broadly at dietary metrics and MCH^10^ or NCDs,^20^, ^27^, ^28^, ^29^, ^30^ but these are more than a decade old and did not also consider both MCH and NCDs together. Consistent with our findings, these previous studies generally found that MCH dietary metrics were not validated against MCH or NCD health outcomes, and that only a modest number of NCD dietary metrics predicted some chronic diseases. Our findings build upon and greatly extend these previous reviews by providing the most extensive contemporary summary of the evidence for associations of dietary metrics with both MCH and NCD outcomes.

Future studies should consider analyses to validate existing dietary metrics with both MCH and NCD outcomes. Additionally, our findings suggest the need for development of novel dietary metrics designed from the start to assess and monitor both MCH and NCDs. Novel dietary metrics could be entirely new, or adaptations of existing dietary metrics and should be developed with several key considerations. First, comprehensive reviews of existing dietary metrics and health outcome relationships can be used to inform the features of new metrics (eg, foods or nutrients, scoring, cutoffs). We found that the four NCD dietary metrics with convincing evidence for associations were comprised of food and nutrient components, included healthy plant foods and dairy, and often included red and processed meat, or sodium. This finding might suggest that these components are important aspects of dietary metrics. However, the validity of most remaining dietary metrics has not been assessed against health outcomes, and it is not known whether dietary metrics without these components are less predictive of validity than those with these components. Second, systematic evaluations to summarise the aetiologic effects and optimal levels of dietary factors for MCH outcomes might provide hypotheses to inform the selection of foods and nutrients in new dietary metrics. In the past 10 years the growing availability of standardised, global dietary data has provided greater clarity on actual food and nutrient intakes,^156^, ^157^ and the relationships between foods and nutrients, and cardiovascular disease, type 2 diabetes^158^ and cancer^159^ are well documented, but similar associations for many MCH outcomes have not been systematically assessed. Third, new dietary metrics need to acknowledge and reconcile potentially opposing effects of some foods and nutrients on MCH and NCD outcomes. For example, animal source foods (eg, meat, fish, eggs, dairy) have been shown to have beneficial effects on micronutrient deficiencies, child growth outcomes, and cognitive function,^148^, ^160^, ^161^ but some of these foods, such as processed meats, are negatively associated with NCDs.^158^, ^159^ Although we did not include sustainability metrics in our Review, an important consideration for future dietary metric development is the incorporation of the measurable effect of different food production systems on the environment in dietary metrics. Lastly, an influential consideration for the widespread adoption of existing dietary metrics by health agencies and governments is likely dependent on cultural appropriateness and ease of collecting and scoring these dietary metrics.

Several challenges have likely limited validation of dietary metrics against both MCH and NCD. Ideally, prospective cohort studies with detailed and accurately measured data on both dietary habits and health outcomes should be leveraged. However, populations with MCH outcomes often do not have strong dietary assessment methods and longitudinal data. Most studies on MCH dietary metrics collect information on dietary intake over the previous 24 hour period because the priority is on identifying trends, comparisons, and rankings of outcomes within and between groups, which is generally of interest in setting and evaluating programme targets, and a longer reference period is not necessarily appropriate for these purposes and might be more difficult to operationalise. Moreover, these studies frequently assess diet qualitatively and the absence of quantitative data limit opportunities to construct and validate existing NCD dietary metrics. Ecological analyses are an alternative study design, which bring their own limitations but could address both MCH and NCD across low-income, middle-income, and high-income countries. In addition to these practical data challenges, one of the most important limitations of previous work might have been the general segregation of diet related MCH versus NCD research and policy.

Our Review has several strengths. We did systematic searches for meta-analyses and narrative reviews of validation against health outcomes, making it less likely that we missed any existing syntheses of dietary metric and health outcome relationships. We included a broad range of health outcomes for MCH and NCDs, informing the modern priorities for the double burden of malnutrition. We evaluated the evidence for health associations independently and in duplicate, including the grading of consistency, reducing the potential for bias.

Limitations should be considered. First, we focused on major MCH and NCD health outcomes, and there might be other relevant health outcomes that were not included, such as cognitive function, bone and joint disorders, and NCD risk factors (hypertension, dyslipidaemia, and glucose control). Second, our systematic reviews for each dietary metric in relation to MCH and NCD health outcomes was limited to published meta-analyses and narrative reviews and did not separately assess individual studies. However, meta-analyses and narrative review are more likely to provide accurate and reliable conclusions compared to single studies, minimising publication bias. Lastly, the evidence for validity came mostly from observational studies and some trials, and these are subject to residual confounding, which could overestimate associations, and measurement error in both diet assessment methods and outcome ascertainment, which might result in an underestimation of the relationships between the dietary metrics and MCH and NCD health outcomes.

In summary, we identified seven international dietary metrics for MCH, none of which have been validated against health outcomes in meta-analyses or narrative reviews, and 12 dietary metrics for NCDs, of which only four have convincing evidence for validation against specific NCD outcomes. These findings highlight important gaps and major opportunities in global analyses of diet quality relating to malnutrition, which are highly relevant to the achievement of the UN SDGs, other global nutrition targets, and corresponding effective policy actions.

## Search strategy and selection criteria

We searched PubMed for articles published between Oct 11, 2000, and April 17, 2020, using the terms “diet quality”, “diet metric”, or “diet score” with “malnutrition”, “undernutrition”, “maternal health”, “child health”, “stunting”, “wasting”, “mortality”, “non-communicable disease”, “chronic disease”, “cardiovascular disease”, “diabetes”, “cancer”, or “obesity” with “meta-analysis”, “systematic review”, or “narrative review”. We used search terms in English but did not apply any language restrictions. We screened articles by title and abstract to identify full-text reports that were relevant to the study aims. We also screened citation lists for these full-text reports to identify other relevant articles. Articles were considered relevant if they reported the relation between dietary metrics and malnutrition. Several previous reviews qualitatively summarised the evidence for dietary metrics in relation to a single health outcome, and other reviews focused on several maternal and child health (MCH) or non-communicable disease (NCD) health outcomes. A few comprehensive reviews of dietary metrics in relation to several MCH or NCD outcomes were completed more than a decade ago, and no review has focused on both MCH and NCD outcomes.
