# Supplementary material for: Defining diet quality: a synthesis of dietary quality metrics and their validity for the double burden of malnutrition
Source: Lancet Planet Health. 2020 Aug 12;4(8):e352–70. doi: 10.1016/S2542-5196(20)30162-5 (PMC7435701; doi:10.1016/S2542-5196(20)30162-5)
Supplement: Supplementary appendix [file mmc1.pdf]

THE LANCET
Planetary Health

Supplementary appendix

This appendix formed part of the original submission and has been peer reviewed. We post it as supplied by the authors.

Supplement to: Miller V, Webb P, Micha R, Mozaffarian D, on behalf of the Global Dietary Database. Defining diet quality: a synthesis of dietary quality metrics and their validity for the double burden of malnutrition. *Lancet Planet Health* 2020; **4:** e352–70.

Appendix

We included dietary metrics with the reported intended use of relating diet to the following MCH and NCD health outcomes:

MCH outcomes: 1) micronutrient adequacy; 2) under-5 mortality; 3) maternal mortality; 4) underweight; 5) stunting; 6) wasting; 7) infectious diseases; 8) diarrheal disease.

NCD outcomes: 1) all-cause mortality; 2) cardiovascular disease; 3) type 2 diabetes; 4) gestational diabetes; 5) total cancer; 6) gastric cancer; 7) colon and rectal cancer; 8) oral cancer; 9) breast cancer; 10) endometrial cancer; 11) kidney cancer; 12) prostate cancer; 13) liver cancer; 14) pancreatic cancer; 15) esophageal cancer; 16) overweight/obese in adults and children; 17) body mass index in adults and children; 18) waist circumference in adults and children.

Search Strategy- Dietary Metrics and Diet-related Health Outcomes

EXPOSURE

(“diet score” [MeSH] or “diet score” [tiab] OR “diet index” [MeSH] OR “diet index” OR “diet metric” [MeSH] OR “diet metric” [tiab] OR “diet quality” [MeSH] OR “diet quality” [tiab] OR “diet diversity” [MeSH] or “diet diversity” [tiab] OR “healthy diet” [tiab] OR “MDD” [tiab] OR “MDD-W” [tiab] OR “minimum dietary diversity” [tiab] OR “IYCMDD” [tiab] OR “WDDS” [tiab] OR “women’s dietary diversity score” [tiab] OR “IDDS” [tiab] OR “individuals dietary diversity score” [tiab] OR “FCS” [tiab] OR “food consumption score” [tiab] OR “HDDS” [tiab] OR “household dietary diversity score” [tiab] OR “FVS” [tiab] OR “food variety score” [tiab] OR “DQI” [tiab] OR “diet quality index” [tiab] OR “DQI-I” [tiab] OR “diet quality index international” [tiab] OR “HDI” [tiab] OR “healthy diet indicator” [tiab] OR “WCRF dietary recommendations” [tiab] OR “MDS” [tiab] OR “Mediterranean diet score” [tiab] OR “aMDS” [tiab] OR “alternative Mediterranean diet score” [tiab] OR “mMDS” [tiab] OR “modified Mediterranean diet score” [tiab] OR “KIDMED” [tiab] OR “HEI” [tiab] OR “healthy eating index” [tiab] OR “aHEI” [tiab] OR “alternative healthy eating index” [tiab] OR “DASH” [tiab] OR “dietary approaches to stop hypertension” [tiab] OR “PURE diet score” [tiab] OR “RFS” [tiab] OR “recommended foods score” [tiab] OR “carbohydrate quality index” [tiab])

AND

OUTCOMES

(“micronutrient” [tiab] OR “micronutrient deficiency” [MeSH] OR “micronutrient deficiency” [tiab] OR “anthropometric” [MeSH] OR “anthropometric” [tiab] OR “weight” [tiab] OR “underweight” [tiab] OR “overweight” [MeSH] OR “overweight” [tiab] OR “obese” [tiab] OR “stunting” [MeSH] OR “stunting” [tiab] OR “wasting” [MeSH] OR “wasting” [tiab] OR “height” [tiab] OR “infectious disease” [MeSH] OR “infectious disease” [tiab] OR “diarrheal disease” [tiab] OR “cardiovascular disease” [MeSH] OR “cardiovascular disease” [tiab] OR “myocardial infarction” [tiab] OR "heart attack” [tiab] OR "heart attacks” [tiab] OR “cardiac death” [tiab] OR “cardiac deaths” [tiab] OR "sudden death"[tiab] OR "sudden deaths" [tiab] OR “myocardial ischemia” [MeSH] OR “myocardial ischemia” [tiab] OR “coronary heart disease”

[MeSH] OR “coronary heart disease” [tiab] OR “coronary artery disease” [MeSH] OR “coronary artery disease” [tiab] OR “stroke” [tiab] OR “strokes” [tiab] OR "cerebrovascular accident"[tiab] OR "cerebrovascular accidents"[tiab] OR “vascular disease” [MeSH] OR “vascular disease” [tiab] OR “diabetes mellitus” [MeSH] OR “diabetes mellitus” [tiab] OR “diabetes” [tiab] OR “diabetes mellitus, Type 2” [MeSH] OR “cancer” [MeSH] OR “cancer” [tiab] OR “gastric cancer” [tiab] OR “stomach cancer” OR “colon cancer” [tiab] OR “rectal cancer” [tiab] OR “oral cancer” [tiab] OR “breast cancer” [tiab] OR “endometrial cancer” [tiab] OR “kidney cancer” [tiab] OR “prostate cancer” [tiab] OR “liver cancer” [tiab] OR “pancreatic cancer” [tiab])

AND

PUBLICATION

(“meta-analysis” [ptyp] OR “meta-analysis” [tiab] OR “meta-analyses” [tiab] OR "systematic review" [tiab] OR "systematic literature review"[tiab] OR "comprehensive review"[tiab] OR "comprehensive literature review” [tiab]**)**

AND

LIMITS

("2000/10/11” [PDat]: "2020/04/17” [PDat]**)**

ALL COMBINED

(“diet score” [MeSH] or “diet score” [tiab] OR “diet index” [MeSH] OR “diet index” OR “diet metric” [MeSH] OR “diet metric” [tiab] OR “diet quality” [MeSH] OR “diet quality” [tiab] OR “diet diversity” [MeSH] or “diet diversity” [tiab] OR “healthy diet” [tiab] OR “MDD” [tiab] OR “MDD-W” [tiab] OR “minimum dietary diversity” [tiab] OR “IYCMDD” [tiab] OR “WDDS” [tiab] OR “women’s dietary diversity score” [tiab] OR “IDDS” [tiab] OR “individuals dietary diversity score” [tiab] OR “FCS” [tiab] OR “food consumption score” [tiab] OR “HDDS” [tiab] OR “household dietary diversity score” [tiab] OR “FVS” [tiab] OR “food variety score” [tiab] OR “DQI” [tiab] OR “diet quality index” [tiab] OR “DQI-I” [tiab] OR “diet quality index international” [tiab] OR “HDI” [tiab] OR “healthy diet indicator” [tiab] OR “WCRF dietary recommendations” [tiab] OR “MDS” [tiab] OR “Mediterranean diet score” [tiab] OR “aMDS” [tiab] OR “alternative Mediterranean diet score” [tiab] OR “mMDS” [tiab] OR “modified

Mediterranean diet score" [tiab] OR "KIDMED" [tiab] OR "HEI" [tiab] OR "healthy eating index" [tiab] OR "aHEI" [tiab] OR "alternative healthy eating index" [tiab] OR "DASH" [tiab] OR "dietary approaches to stop hypertension" [tiab] OR "PURE diet score" [tiab] OR "RFS" [tiab] OR "recommended foods score" [tiab] OR "carbohydrate quality index" [tiab]) **AND** ("micronutrient" [tiab] OR "micronutrient deficiency" [MeSH] OR "micronutrient deficiency" [tiab] OR "anthropometric" [MeSH] OR "anthropometric" [tiab] OR "weight" [tiab] OR "underweight" [tiab] OR "overweight" [MeSH] OR "overweight" [tiab] OR "obese" [tiab] OR "stunting" [MeSH] OR "stunting" [tiab] OR "wasting" [MeSH] OR "wasting" [tiab] OR "height" [tiab] OR "infectious disease" [MeSH] OR "infectious disease" [tiab] OR "diarrheal disease" [tiab] OR "cardiovascular disease" [MeSH] OR "cardiovascular disease" [tiab] OR "myocardial infarction" [tiab] OR "heart attack" [tiab] OR "heart attacks" [tiab] OR "cardiac death" [tiab] OR "cardiac deaths" [tiab] OR "sudden death"[tiab] OR "sudden deaths" [tiab] OR "myocardial ischemia" [MeSH] OR "myocardial ischemia" [tiab] OR "coronary heart disease" [MeSH] OR "coronary heart disease" [tiab] OR "coronary artery disease" [MeSH] OR "coronary artery disease" [tiab] OR "stroke" [tiab] OR "strokes" [tiab] OR "cerebrovascular accident"[tiab] OR "cerebrovascular accidents"[tiab] OR "vascular disease" [MeSH] OR "vascular disease" [tiab] OR "diabetes mellitus" [MeSH] OR "diabetes mellitus" [tiab] OR "diabetes" [tiab] OR "diabetes mellitus, Type 2" [MeSH] OR "cancer" [MeSH] OR "cancer" [tiab] OR "gastric cancer" [tiab] OR "stomach cancer" OR "colon cancer" [tiab] OR "rectal cancer" [tiab] OR "oral cancer" [tiab] OR "breast cancer" [tiab] OR "endometrial cancer" [tiab] OR "kidney cancer" [tiab] OR "prostate cancer" [tiab] OR "liver cancer" [tiab] OR "pancreatic cancer" [tiab]) **AND (**"meta-analysis" [ptyp] OR "meta-analysis" [tiab] OR "meta-analyses" [tiab] OR "systematic review" [tiab] OR "systematic literature review"[tiab] OR "comprehensive review"[tiab] OR "comprehensive literature review" [tiab]**) AND (**"2000/10/11" [PDat]: "2020/04/17" [PDat]**)**

Table S1. Dietary metrics considered and excluded from the review.

Metric
Adolescent micronutrient quality index
American heart association (AHA) diet and lifestyle recommendation (AHA DLR)
Australian dietary guideline index for children and adolescents (DGI-CA)
Australian guide to healthy eating (AGHE)
Australian recommended food score (ARFS)
Australian recommended food score for preschoolers (ARFS-P)
Baltic sea diet score (BSDS)
Diet variety score (DVS)
Diet variety score for recommended foods
Dietary guideline index (DQI)
Dietary quality score (DQS)
Dutch healthy diet index (DHD-index)
Elderly dietary index (EDI)
Food pyramid index (FPI)
Food-based quality index
Foods E-KINDEX
Framingham nutritional risk score (FNRS)
French program national nutrition sante-guideline score (PNNS-GS)
German food pyramid index
Global hunger index
GLOPAN elements of high-quality diet
Healthy food index
Healthy nordic food index (HNFI)
Infant and child feeding index (ICFI)
Low carbohydrate diet score (LCDS)
Low carbohydrate high protein diet score (LCHP)
Mean adequacy ratio (MAR) or nutrient adequacy ratio (NAR)
Mozambique diet assessment tool household level
Non-recommended food score (NRFS)
Nutrient rich food (NRF) index
Overall dietary index-revised (ODI-R)
Percent energy from ultra-processed foods
Portfolio diet
Prime diet quality score
Recommended food and behavior score
Recommended food and beverage score
U.S healthy food diversity (HFD) index
WHO 5 keys to a healthy diet
WHO fruit and vegetable indicator, STEPS version
WHO healthy diet

Table S2. Dietary metrics validity and reliability.

Dietary metric	Validity and reliability	Strengths of validation studies	Limitations of validation studies
**Dietary metrics for the assessment of maternal and child health**			
Dietary Diversity Score (DDS)(1-3)	**Assessment of validity**<br>Narrative review<br><br>**Validated against disease**<br>See Figure 3<br><br>**Reliability**<br>Not demonstrated	• Moderate to large sample size for outcomes.<br>• Considered a minimum quantity when assessing intake (≥10g).<br>• Not assessed for children under the age of 24 months for some outcomes.	• Not validated for micronutrient adequacy and disease outcomes (stunting, wasting, diarrhea, underweight, overweight/obesity, cardiovascular disease, type II diabetes, cancer) in published meta-analyses and narrative reviews.
World Food Programme's Food Consumption Score (FCS)(4-6)	**Assessment of validity**<br>Paper/report on metric development<br><br>**Validated against other metrics**<br>Validated against the Darfur Dietary Diversity Metrics, and the Household Dietary Diversity Score (HDDS) for per capita household energy consumption in a sample of 2,244 households from 3 countries (Burundi, Haiti and Sri Lanka). The FCS provided lower estimates of food insecurity compared to classification by calorie consumption per capita. A universal cutoff was not found.<br><br>**Reliability**<br>Not demonstrated	• More representative of usual intake.<br>• Includes quantitative data for some foods and nutrients.<br>• Food group weights selected to reflect nutrient density (calories, and macronutrient and micronutrient content).	• Not individual level.<br>Complex scoring with different weights for each component.<br>• Small number of datasets from various settings used<br>Small sample size for some datasets.<br>• Not validated for micronutrient adequacy and disease outcomes (stunting, wasting, diarrhea, underweight, overweight/obesity, cardiovascular disease, type II diabetes, cancer) in published meta-analyses and narrative reviews.
Food Variety Score (FVS)(1, 7, 8)	**Assessment of validity**<br>Narrative review<br><br>**Validated against disease**<br>See Figure 3<br><br>**Reliability**<br>Not demonstrated	• Moderate sample size for some outcomes.<br>• Not assessed for children under the age of 12 months.	• Small sample size for some outcomes.<br>• No quantitative information about food and nutrient intake.<br>• Not validated for micronutrient adequacy and disease outcomes (stunting, wasting, diarrhea, underweight, overweight/obesity, cardiovascular disease, type II diabetes, cancer) in published meta-analyses and narrative reviews.

Household Dietary Diversity Score (HDDS)(9-11)	**Assessment of validity**<br>Paper/report on metric development<br><br>**Validated against other metrics**<br>10 datasets from 10 countries (N=28,498) were used for the development and validation. Associations between HDDS and 4 metrics of household food access were considered: 1) per capita consumption; 2) per capita caloric availability; 3) per capita caloric availability from staples (cereals and cereal products); 4) per capita caloric availability from non-staples. On average, a 1% increase in HDDS was associated with a 1% increase in household per capita consumption, a 0.7% increase in household per capita caloric availability, a 0·5% increase in household per capita caloric availability from staples, and a 1·4% increase in household per capita availability from non-staples.<br><br>**Reliability**<br>Not demonstrated	• Included data from 10 countries and 3 continents in validation study,	• Not individual level.<br>• Small sample size for some datasets.<br>• No quantitative information about food and nutrient intake.<br>• Not validated for micronutrient adequacy and disease outcomes (stunting, wasting, diarrhea, underweight, overweight/obesity, cardiovascular disease, type II diabetes, cancer) in published meta-analyses and narrative reviews.
Infant and Young Child Minimum Dietary Diversity (IYCMDD)(12, 13)	**Assessment of validity**<br>Paper/report on metric development<br><br>**Validated against micronutrients**<br>Several food group metrics (FGI) were tested (FGI-7, FGI-8), with and without restricted minimum portions (1-g minimum and 10-g minimum) using data from 8 countries in Africa, Asia and Latin-America. In total, the analysis included 8,510 child-days of intake for breastfed children and 1,504 child-days of data for non-breastfed children. Standardized methodology assessed the association between the FGIs and mean micronutrient density adequacy (MMDA; 0·50 and 0·75) of the diet for the nutrients.	• Applicable to both breastfed and non-breastfed children.<br>• Included data from 8 countries and 3 continents in validation study.	• Short reference period.<br>• No quantitative information about food and nutrient intake.<br>• Small number of datasets from various settings used.<br>• Small sample size for some datasets.<br>• Not validated for micronutrient adequacy and disease outcomes (wasting, diarrhea, underweight, overweight/obesity, cardiovascular disease, type II diabetes, cancer) in published meta-analyses and narrative reviews.

	Sensitivity and specificity analyses failed to identify a cutoff that performed best across all countries. The cutoff ≥ 4 was selected based on stakeholder discussions.<br><br>**Validated against disease**<br>See Figure 3<br><br>**Reliability**<br>Not demonstrated		
Minimum Dietary Diversity for Women (MDD-W)(14, 15)	**Assessment of validity**<br>Paper/report on metric development<br><br>**Validated against micronutrients**<br>Data from 6 countries in Africa, Asia and Latin-America were used for the development and validation (N=4,166 women). Standardized methodology assessed the association between the FGIs and mean probability of adequacy (MPA; 0·50, 0·60 and 0·70) of the diet for the nutrients.<br>Sensitivity and specificity analyses were moderate to poor at the individual level, but results were consistent enough to recommend the use of a dichotomous FGI for global use. The cutoff ≥ 5 for FGI-9 and FGI-10 was selected based on stakeholder discussions.<br><br>**Reliability**<br>Not demonstrated	• Included data from 6 countries and 3 continents in validation study.	• Short reference period.<br>• No quantitative information about food and nutrient intake.<br>• Small number of datasets from various settings used<br>Small sample size for some datasets.<br>• Unable to examine pregnant women separately,<br>• High exclusion rate for 1 dataset and moderate for 2 other datasets.<br>• Not validated for micronutrient adequacy and disease outcomes (stunting, wasting, diarrhea, underweight, overweight/obesity, cardiovascular disease, type II diabetes, cancer) in published meta-analyses and narrative reviews.
Women's Dietary Diversity Score (WDDS) and Individual Dietary Diversity Score (IDDS)(10, 15)	**Assessment of validity**<br>Paper/report on metric development<br><br>**Validated against micronutrients**<br>Several food group metrics (FGI) were tested (FGI-6, FGI-9, FGI-13, FGI-21), with and without restricted minimum portions (1-g minimum and 15-g minimum) using data from 5 countries in Africa and Asia. In total, the analysis included 4,166 women. Standardized	• Included data from 5 countries and 2 continents in validation study.	• Short reference period.<br>• No quantitative information about food and nutrient intake.<br>• Small number of datasets from various settings used.<br>• Small sample size for some datasets.<br>• Unable to examine pregnant women separately.<br>• High exclusion rate for 1 dataset and moderate for 2 other datasets.

	methodology assessed the association between the FGIs and mean probability of adequacy (MPA; 0·50, 0·60 and 0·70) of the diet for the nutrients.<br><br>There most disaggregated FGIs performed were more highly correlated with micronutrient adequacy of the diet in most countries. No single FGI performed consistently better across all 5 settings, but the FGI-9 was adopted by FAO in its guidance document.<br>The IDDS has not been validated.<br><br>**Reliability**<br>Not demonstrated		• Not validated for micronutrient adequacy and disease outcomes (stunting, wasting, diarrhea, underweight, overweight/obesity, cardiovascular disease, type II diabetes, cancer) in published meta-analyses and narrative reviews.
**Dietary metrics for the assessment of non-communicable disease risk**			
Alternative Healthy Eating Index (AHEI-2010)	**Assessment of validity**<br>Meta-analysis<br><br>**Validated against disease**<br>See Table 3 and Figure 3<br><br>**Reliability**<br>Not demonstrated	• More representative of usual intake.<br>• Includes quantitative data for some foods and nutrients.	• Not validated for micronutrient adequacy and some disease outcomes (stunting, wasting, underweight, overweight/obesity, some cancer subtypes) in published meta-analyses and narrative reviews.
Dietary Approaches to Stop Hypertension (DASH)(16)	**Assessment of validity**<br>Meta-analysis<br><br>**Validated against disease**<br>See Table 3 and Figure 3<br><br>**Reliability**<br>Not demonstrated	• More representative of usual intake.<br>• Includes quantitative data for some foods and nutrients.	• Scoring relative to sample distribution.<br>• Not validated for micronutrient adequacy and some disease outcomes (stunting, wasting, underweight, overweight/obesity in children, some cancer subtypes) in published meta-analyses and narrative reviews.<br>• Use limited to populations consuming diets similar to the DASH diet pattern.
Dietary Guidelines for Americans Adherence Index (DGAI)(17-22)	**Assessment of validity**<br>Paper/report on metric development<br><br>**Validated against foods/nutrients**<br>In 3,323 participants enrolled in the Framingham Heart Study (FHS) Offspring Cohort correlations were computed between the total DGAI score, the food intake and healthy	• More representative of usual intake.<br>• Includes quantitative data for some foods and nutrients.	• Not validated for micronutrient adequacy and disease outcomes (stunting, wasting, diarrhea, underweight, overweight/obesity, cardiovascular disease, type II diabetes, cancer) in published meta-analyses and narrative reviews.

	subscores and the individual components. All nutrients except for vitamin B12 and sodium were significantly associated with the DGAI score. Positive correlations were found between the DGAI score and intakes of carbohydrate, fiber, protein (n-3) fatty acids, vitamins C and E, folate, calcium and positive. Negative correlations were found between the DGAI score and intakes of total fat, saturated fat, mono- and polyunsaturated fat, trans fat, and cholesterol. The spearman correlation coefficients for the total DGAI score and components ranged from 0.07 (sodium) to 0.74 (variety of fruits and vegetables). The subscores were highly correlated with the total DGAI score and modestly intercorrelated.<br><br>**Reliability**<br>Not demonstrated		• Use limited to populations consuming diets similar to the American Dietary Guideline recommendations.
Dietary Inflammatory Index (DII)(23-25)	**Assessment of validity**<br>Meta-analysis and paper/report on metric development<br><br>**Validated against disease**<br>See Table 3 and Figure 3<br><br>**Validated against other metrics**<br>In 500 participants enrolled in the Seasonal Variation in Blood Cholesterol Study (SEASONS) the third tertile of DII compared to the first tertile was associated with 47% to 61% increased odds of having elevated CRP (>3mg/L).<br>In 2600 participants in Women's Health Initiative Observational study, a more pro-inflammatory diet was associated with higher IL-6, hs-CRP, TNFalpha R2, and an overall biomarker score derived from a combination of the 3 biomarkers.	• Validated against disease and biomarkers.<br>• More representative of usual intake.<br>• Includes quantitative data for some foods and nutrients.	• Not validated for micronutrient adequacy and disease outcomes (stunting, wasting, diarrhea, underweight, overweight/obesity, cardiovascular disease, type II diabetes, most cancers) in published meta-analyses and narrative reviews.

	**Reliability**<br>Not demonstrated		
Diet Quality Index International (DQI-I)(26-28)	**Assessment of validity**<br>Narrative review<br><br>**Validated against disease**<br>See Figure 3<br><br>**Reliability**<br>Not demonstrated	• More representative of usual intake (multiple 24-hr reference period and FFQ used).<br>• Includes quantitative data for some foods and nutrients.	• Justification for component weights not provided.<br>• Not validated for micronutrient adequacy and disease outcomes (stunting, wasting, diarrhea, underweight, overweight/obesity, cardiovascular disease, type II diabetes, most cancers) in published meta-analyses and narrative reviews.
Healthy Eating Index (HEI-2010)(29-33)	**Assessment of validity**<br>Meta-analysis<br><br>**Validated against disease**<br>See Table 3 and Figure 3<br><br>**Validated against foods/nutrients**<br>NHANES data from 2001-2002 was used to evaluate HEI-2005, 2003-2004 for HEI-2010 and 2011-2012 for HEI-2015. For content validity, all the food items described in the dietary guidelines were included as components in the HEI. For construct validity, the sample menus scored moderately on the HEI, and one-day scores between smoker and non-smokers were significantly different indicating that the HEI can distinguish between groups with known differences in diet quality. Additionally, the Pearson's correlation coefficients of the total HEI and component scores with energy showed that the HEI can assess diet quality independently of diet quantity.<br><br>**Reliability**<br>Cronbach's coefficient alpha was used to assess internal consistency. Most components were positively correlated with the total HEI score, except for sodium and dairy.	• More representative of usual intake.<br>• Includes quantitative data for some foods and nutrients.<br>• Assesses diets on a per 1,000 calories basis to control for diet quantity.<br>• Applicable to pregnant and lactating women.	• Not validated for micronutrient adequacy and some disease outcomes (stunting, wasting, underweight, overweight/obesity in children, type II diabetes, some cancer subtypes) in published meta-analyses and narrative reviews.<br>• Not applicable to children <2 years.<br>• Validity markedly different for some ethnic and cultural groups compared to the American norm.

Mediterranean Diet Quality Index for Children and Teenagers (KIDMED)(34-41)	**Assessment of validity**<br>Narrative review<br><br>**Validated against disease**<br>See Figure 3<br><br>**Reliability**<br>A study of 276 college students found moderate to excellent test-retest reliability (κ=0.597, p<0.001)(38).	• More representative of usual intake.<br>• Includes quantitative data for some foods and nutrients.	• Scoring relative to sample distribution.<br>• Not validated for micronutrient adequacy and some disease outcomes (stunting, wasting, underweight, overweight/obesity in adults, type II diabetes, some cancer subtypes) in published meta-analyses and narrative reviews.<br>• Use limited to populations consuming diets similar to the Mediterranean diet pattern.
Mediterranean Diet Score (MED)(42-45)	**Assessment of validity**<br>Meta-analysis<br><br>**Validated against disease**<br>See Table 3 and Figure 3<br><br>**Reliability**<br>Not demonstrated	• More representative of usual intake.<br>• Includes quantitative data for some foods and nutrients.	• Scoring relative to sample distribution.<br>• Not validated for micronutrient adequacy and some disease outcomes (stunting, wasting, underweight, overweight/obesity in children, some cancer subtypes) in published meta-analyses and narrative reviews.<br>• Use limited to populations consuming diets similar to the Mediterranean diet pattern.
Prospective Urban Rural Epidemiology (PURE) Diet Score(46)	**Assessment of validity**<br>Not validated against disease in a meta-analysis or narrative review.<br><br>Among 218,000 adults from 50 countries included in 4 international studies (PURE, ONTARGET, INTERHEART, and INTERSTROKE) the association between the PURE diet score and risk of mortality, cardiovascular disease, myocardial infarction and stroke were examined. The PURE diet score was inversely associated with mortality, cardiovascular disease, myocardial infarction and stroke.<br><br>**Reliability**<br>Not demonstrated	• More representative of usual intake.<br>• Includes quantitative data for foods.<br>• Included data from 50 countries in validation study.	• Overall quintiles used to derive component scores which may group entire countries or regions into the same quintile.<br>• Not validated for micronutrient adequacy and some disease outcomes (stunting, wasting, underweight, overweight/obesity in adults, type II diabetes, some cancer subtypes) in published meta-analyses and narrative reviews.
Recommended foods score (RFS)(47-51)	**Assessment of validity**<br>Narrative review<br><br>**Validated against disease**<br>See Figure 3	• More representative of usual intake.<br>• Includes quantitative data for some foods and nutrients.	• Scoring relative to sample distribution.<br>• Not validated for micronutrient adequacy and some disease outcomes (under- or overweight and obesity, type II diabetes, cancer).

	**Reliability**<br>Not demonstrated		
WHO Healthy Diet Indicator (WHO-HDI)(52-56)	**Assessment of validity**<br>Narrative review<br><br>**Validated against disease**<br>See Figure 3<br><br>**Reliability**<br>Not demonstrated	• More representative of usual intake.<br>• Includes quantitative data for some foods and nutrients.	• Short Reference period.<br>• Not validated for micronutrient adequacy and some disease outcomes (stunting, wasting, underweight, overweight/obesity in adults, type II diabetes, some cancer subtypes) in published meta-analyses and narrative reviews.<br>• Use limited to European populations.
World Cancer Research Fund and American Institute for Cancer Research (WCRF/AICR) dietary recommendations(57-59)	**Assessment of validity**<br>Narrative review<br><br>**Validated against disease**<br>See Figure 3<br><br>**Reliability**<br>Not demonstrated	• More representative of usual intake.<br>• Includes quantitative data for foods.<br>• Included data from 6 countries in validation study.	• Limited discrimination between score categories.<br>• Not validated for micronutrient adequacy and some disease outcomes (stunting, wasting, underweight, overweight/obesity in adults, type II diabetes, some cancer subtypes) in published meta-analyses and narrative reviews.

Validity against foods and/or nutrients considered the correlation between the dietary metric and individual foods or nutrients.
Validity against other metrics examined the association between the dietary metric and other non-dietary metrics such as biomarkers, and food insecurity indicators.
Both validity against foods and/or nutrients were extracted from papers and technical reports.
Validity against health outcomes was assessed as the consistency of the association between the dietary metric and health outcome.
Reliability was defined as repeated measure validity.
Dietary Diversity Score (DDS) was classified as a maternal and child health dietary metric because it has primarily been used for this purpose despite being originally developed for chronic diseases.
The Infant and Young Child Minimum Dietary Diversity (IYCMDD) metric is specific to infants and young child, and the Minimum Dietary Diversity for Women (MDD-W) and Women's Dietary Diversity Score (WDDS) are specific to women of reproductive age (15 to 49 years of age).

Table S3. Estimates of etiologic effects of dietary metrics and risk of health outcomes.

AHEI									
**Outcome**	**Search date**	**Studies included**	**Source**	**No. of subjects**	**Countries**	**Unit**	**RR (95% CI)**	**I^2**	**P for heterogeneity**
All-cause mortality	May 10, 2014	3	Schwingshackl et al., 2015(60)	541,978	USA, Great Britain	High vs. low	0·77 (0·75-0·79)	42%	0·16
	December 14, 2015	9	Onvani et al., 2017(61)	826,082	USA, China, United Kingdom	High vs. low	0·77 (0·76-0·78)	N.R.	N.R.
	May 15, 2017	7	Schwingshackl et al., 2018(62)	975,639	USA, China, Great Britain	High vs. low	0·76 (0·74-0.79)	71%	0·003
All-cause mortality among cancer survivors	May 15, 2017	3	Schwingshackl et al., 2018(62)	9,508	USA	High vs. low	0·85 (0·70-1·03)	65%	0·03
Cardiovascular disease	May 10, 2014	6	Schwingshackl et al., 2015(60)	854,064	USA, Great Britain	High vs. low	0·74 (0·72-0·77)	10%	0·35
	May 15, 2017	13	Schwingshackl et al., 2018(62)	1,296,276	USA, China, United Kingdom	High vs. low	0·75 (0·72-0·77)	39%	0·05
Cardiovascular mortality	December 14, 2015	7	Onvani et al., 2017(61)	820,778	USA, China, United Kingdom	High vs. low	0.74 (0.71-0.78)	N.R.	N.R.
Type II diabetes	May 10, 2014	6	Schwingshackl et al., 2015(60)	409,228	USA, Denmark, France, Germany, Italy, Spain, Sweden, United Kingdom, the Netherlands	High vs. low	0·77 (0·68-0·86)	80%	0·001
	December 31, 2015	5	Jannasch et al., 2017(63)	373,414	USA, Denmark, France, Germany, Italy, Spain, Sweden, United Kingdom, the Netherlands	High vs. low	0·79 (0·70-0·89)	88%	<0·001
	May 15, 2017	9	Schwingshackl et al., 2018(62)	605,077	USA, Denmark, France, Germany, Italy, Spain, Sweden, United Kingdom, the Netherlands	High vs. low	0·80 (0·74-0·86)	76%	<0·001
Cancer	May 10, 2014	7	Schwingshackl et al., 2015(60)	1,546,234	USA, Great Britain	High vs. low	0·89 (0·84-0·94)	72%	0·002
	May 15, 2017	18	Schwingshackl et al., 2018(62)	3,013,168	USA, Great Britain, China, Australia	High vs. low	0·88 (0·85-0·91)	54%	0·001
Cancer mortality	December 14, 2015	8	Onvani et al., 2017(61)	821,984	USA, China, United Kingdom	High vs. low	0·85 (0·83-0·88)	N.R.	N.R.
	June 2017	9	Milajerdi et al., 2018(64)	964,740	USA, England	High vs. low	0·90 (0·85-0·95)	62%	0·003

Cancer mortality among cancer survivors	May 15, 2017	3	Schwingshackl et al., 2018(62)	9,508	USA	High vs. low	0·95 (0·79-1·13)	20%	0·29
**DASH**									
**Outcome**	**Meta-analysis search date**	**Studies included**	**Source**	**No. of subjects**	**Countries**	**Unit of exposure**	**RR (95% CI)**	**I^2**	**P for heterogeneity**
All-cause mortality	May 15, 2017	8	Schwingshackl et al., 2018(62)	1,353,039	USA, China, Denmark, France, Germany, Greece, Italy, the Netherlands, Spain, Sweden, Norway, United Kingdom	High vs. low	0·80 (0·79-0·82)	9%	0·36
All-cause mortality among cancer survivors	May 15, 2017	3	Schwingshackl et al., 2018(62)	9,508	USA	High vs. low	0·94 (0·82-1·08)	27%	0·25
Cardiovascular disease	January 2012	2	Salehi-Abargouei et al., 2013(65)	55,820	USA	High vs. low	0·79 (0·71-0·88)	N.R.	N.R.
	May 10, 2014	11	Schwingshackl et al., 2015(60)	783,732	USA, Taiwan and Italy	High vs. low	0·80 (0·76-0·85)	30%	0·16
	May 15, 2017	18	Schwingshackl et al., 2018(62)	1,745,815	USA, Taiwan, China, United Kingdom, Denmark, France, Germany, Greece, Italy, the Netherlands, Spain, Sweden and Norway	High vs. low	0·80 (0·77-0·84)	49%	0·006
Coronary heart disease	January 2012	3	Salehi-Abargouei et al., 2013(65)	144,337	USA	High vs. low	0·79 (0·71-0·88)	0%	0·583
Coronary artery disease	June 2019	7	Yang et al., 2019(66)	377,725	USA, United Kingdom, the Netherlands	High vs. low	0·82 (0·78-0·87)	0%	0·53
Total stroke	January 2012	3	Salehi-Abargouei et al., 2013(65)	150,191	USA, Italy	High vs. low	0·81 (0·72-0·92)	0%	0·912
	May 2018	11	Feng et al., 2018(67)	474,228	USA, Hong Kong, Taiwan, Italy, Sweden, Germany, United Kingdom, the Netherlands	High vs. low	0·88 (0·83-0·93)	4%	N.R.
Type II diabetes	August 31, 2013	3	Esposito et al., 2014(68)	46,890	USA	High vs. low	0·73 (0·65-0·83)	0%	<0·001
	May 10, 2014	4	Schwingshackl et al., 2015(60)	63,044	USA, Denmark, France, Germany, Italy, Spain,	High vs. low	0·79 (0·66-0·95)	66%	<0·001

					Sweden, United Kingdom, the Netherlands				
	December 31, 2015	5	Jannasch et al., 2017(63)	158,408	USA, Denmark, France, Germany, Italy, Spain, Sweden, United Kingdom, the Netherlands	High vs. low	0·82 (0·72-0·92)	62%	0·03
	May 15, 2017	8	Schwingshackl et al., 2018(62)	258,893	USA, Denmark, France, Germany, Italy, Spain, Sweden, United Kingdom, the Netherlands	High vs. low	0·80 (0·74-0·86)	61%	0·01
Cancer	May 10, 2014	3	Schwingshackl et al., 2015(60)	1,117,410	USA	High vs. low	0·80 (0·77-0·83)	0%	0·61
	May 15, 2017	14	Schwingshackl et al., 2018(62)	2,987,645	USA, Sweden, China, Denmark, France, German, Greece, Italy, Netherlands, Spain, Norway, United Kingdom	High vs. low	0·82 (0·80-0·86)	48%	0·007
Cancer mortality	June 2017	9	Milajerdi et al., 2018(64)	1,281,190	USA, England, Colombia	High vs. low	0·85 (0·79-0·91)	82%	0·000
	July 2018	9	Ali Mohsenpour et al., 2019(69)	1,414,944	USA, China, Denmark, France, Germany, Greece, Italy, the Netherlands, Norway, Spain, United Kingdom, Sweden, Singapore	High vs. low	0·84 (0·81-0·86)	13%	0·323
Cancer mortality among cancer survivors	May 15, 2017	3	Schwingshackl et al., 2018(62)	9,508	USA	High vs. low	0·93 (0·79-1·10)	0%	0·73
Colorectal cancer	July 2018	4	Ali Mohsenpour et al., 2019(69)	890,755	USA	High vs. low	0·79 (0·75-0·83)	0%	0·979
	April 2019	6	Mohseni et al., 2020(70)	836,218	USA, Canada	High vs. low	0·81 (0·75-0·88)	54%	0·017
	September 2019	5	Tangestani et al., 2020(71)	819,949	China, USA	High vs. low	0·80 (0·74-0·85)	32%	0·137
Colon cancer	July 2018	2	Ali Mohsenpour et al., 2019(69)	624,587	USA	High vs. low	0·80 (0·74-0·87)	0%	0·922
Rectal cancer	July 2018	2	Ali Mohsenpour et al., 2019(69)	624,287	USA	High vs. low	0·84 (0·74-0·96)	16%	0·274
Weight loss in adults	December 2015	10	Soltani et al., 2016(72)	1,291	USA, Australia, Iran	DASH diet vs. control diet	-1·42 (-2·03 to -0·82)	71%	<0·001

	September 2018	14	Ge et al., 2020(73)	1,110	USA, France, Australia, Brazil, Iran	DASH diet vs. usual diet	-3·63 (-4·76 to 2·52)	N.R.	N.R.
Body mass index in adults	December 2015	6	Soltani et al., 2016(72)	1,157	USA, Iran and China	DASH diet vs. control diet	-0·42 (-0·64 to -0·20)	82%	0·01
Waist circumference in adults	December 2015	2	Soltani et al., 2016(72)	511	USA and Iran	DASH diet vs. control diet	-1·05 (-1·61 to -0·49)	80%	<0·001
**DDS**									
**Outcome**	**Meta-analysis search date**	**Studies included**	**Source**	**No. of subjects**	**Countries**	**Unit of exposure**	**RR (95% CI)**	**I^2**	**P for heterogeneity**
Cancer mortality	June 2017	2	Milajerdi et al., 2018(64)	12,080	USA, Taiwan	High vs. low	1·03 (0·59-1·82)	63%	0·068
**DII**									
**Outcome**	**Meta-analysis search date**	**Studies included**	**Source**	**No. of subjects**	**Countries**	**Unit of exposure**	**RR (95% CI)**	**I^2**	**P for heterogeneity**
All-cause mortality	N.R.	5	Shivappa et al., 2017(74)	99,147	United Kingdom, USA, Sweden, France	High vs. low	1·04 (1·03-1·05)	53%	0·074
Cardiovascular mortality	N.R.	4	Shivappa et al., 2017(74)	91,260	United Kingdom, USA, Sweden	High vs. low	1·05 (1·03-1·07)	15%	0·319
Cancer mortality	N.R.	5	Shivappa et al., 2017(74)	99,142	United Kingdom, USA, Sweden, France	High vs. low	1·05 (1·03-1·07)	30%	0·22
Breast cancer	February 2017	5	Zahedi et al., 2018(75)	279,402	USA, Sweden, France	High vs. low	1·04 (0·98-1·10)	31%	0·218
Gastric cancerα	December 2018	3	Du et al., 2019(76)	2,118	Italy, Korea, Iran	Low vs. high	2·11 (1·41-3·15)	41%	0·19
**DQI**									
**Outcome**	**Meta-analysis search date**	**Studies included**	**Source**	**No. of subjects**	**Countries**	**Unit of exposure**	**RR (95% CI)**	**I^2**	**P for heterogeneity**
Cancer mortality	June 2017	5	Milajerdi et al., 2018(64)	599,041	Sweden, USA, Spain, England	High vs. low	0·91 (0·89-0·93)	2%	0·420
**HEI**									
**Outcome**	**Meta-analysis search date**	**Studies included**	**Source**	**No. of subjects**	**Countries**	**Unit of exposure**	**RR (95% CI)**	**I^2**	**P for heterogeneity**

All-cause mortality	May 10, 2014	2	Schwingshackl et al., 2015(60)	510,434	USA	High vs. low	0·78 (0·76-0·79)	0%	0·86
	December 14, 2015	7	Onvani et al., 2017(61)	741,289	USA	High vs. low	0·78 (0·76-0·79)	N.R.	N.R.
	May 15, 2017	8	Schwingshackl et al., 2018(62)	1,328,413	USA, Denmark, France, Germany, Greece, Italy, the Netherlands, Norway, Spain, Sweden, United Kingdom	High vs. low	0·78 (0·76-0·80)	37%	0·11
All-cause mortality among cancer survivors	May 15, 2017	5	Schwingshackl et al., 2018(62)	12,040	USA	High vs. low	0·85 (0·75-0·96)	26%	0·24
Cardiovascular disease	May 10, 2014	5	Schwingshackl et al., 2015(60)	782,440	USA and Italy	High vs. low	0·82 (0·79-0·85)	0%	0·53
	May 15, 2017	11	Schwingshackl et al., 2018(62)	1,600,121	USA, Denmark, France, Germany, Greece, Italy, the Netherlands, Norway, Spain, Sweden, United Kingdom	High vs. low	0·79 (0·77-0·82)	16%	0·28
Cardiovascular mortality	December 14, 2015	5	Onvani et al., 2017(61)	740,455	USA	High vs. low	0·79 (0·76-0·83)	N.R.	N.R.
Type II diabetes	May 15, 2017	3	Schwingshackl et al., 2018(62)	303,213	USA	High vs. low	0·87 (0·82-0·93)	61%	0·05
Cancer	May 10, 2014	11	Schwingshackl et al., 2015(60)	3,549,700	USA	High vs. low	0·84 (0·78-0·89)	79%	<0·001
	May 15, 2017	21	Schwingshackl et al., 2018(62)	5,048,954	USA, Denmark, France, Germany, Greece, Italy, the Netherlands, Norway, Spain, Sweden, United Kingdom	High vs. low	0·83 (0·79-0·87)	73%	<0·001
Cancer mortality	December 14, 2015	6	Onvani et al., 2017(61)	741,091	USA, China	High vs. low	0·80 (0·76-0·83)	N.R.	N.R.
	June 2017	2	Milajerdi et al., 2018(64)	964,740	USA	High vs. low	0·82 (0·69-0·98)	0%	0·758
Cancer mortality among cancer survivors	May 15, 2017	5	Schwingshackl et al., 2018(62)	12,040	USA	High vs. low	0·84 (0·73-0·97)	18%	0·30
**IYCMDD**									
**Outcome**	**Meta-analysis search date**	**Studies included**	**Source**	**No. of subjects**	**Countries**	**Unit of exposure**	**RR (95% CI)**	**I^2**	**P for heterogeneity**
Stuntingα	November 2017	5	Berhe et al., 2019(77)	N.R.	Ethiopia	<4 score vs. ≥4 score	1·95 (1·31-2·92)	72%	0·006
**MED**									

Outcome	Meta-analysis search date	Studies included	Source	No. of subjects	Countries	Unit of exposure	RR (95% CI)	I^2	P for heterogeneity
All-cause mortality	N.R.	7	Bonaccio et al., 2018(78)	11,738	Australia, Greece, Sweden, United Kingdom, Italy, Belgium, Denmark, France, the Netherlands, Portugal, Spain, Switzerland	1-point increase	0·95 (0·93-0·96)	0%	0·47
Cardiovascular disease	June 2014	13	Grosso et al., 2017(79)	275,162	Greece, Spain, USA, Italy, Germany, Sweden	High vs. low	0·73 (0·66-0·80)	36%	N.R.
	August 2016	11	Rosato et al., 2017(80)	758,280	USA, Spain, Sweden, United Kingdom, the Netherlands, Italy, Finland	High vs. low	0·81 (0·74-0·88)	80%	<0·0001
	May 7, 2018	8	Becerra-Tomas et al., 2019(81)	53,508	Greece, Spain, Italy, USA, United Kingdom, Denmark	High vs. low	0·88 (0·74-1·03)	53%	0·04
Cardiovascular mortality	June 2014	13	Grosso et al., 2017(79)	767,232	USA, Spain, Australia, Greece, Italy, Sweden	High vs. low	0·75 (0·68-0·83)	75%	N.R.
	May 7, 2018	21	Becerra-Tomas et al., 2019(81)	883,878	USA, United Kingdom, Denmark, Spain, Switzerland, Italy, Australia, Sweden	High vs. low	0·79 (0·77-0·82)	0%	0·64
Coronary heart disease	June 2014	4	Grosso et al., 2017(79)	139,893	Spain, USA, Greece	High vs. low	0·72 (0·60-0·86)	N.R.	N.R.
	August 2016	11	Rosato et al., 2017(80)	379,473	USA, Spain, Sweden, the Netherlands, Greece, Italy, Finland	High vs. low	0·70 (0·62-0·80)	45%	0·06
	May 7, 2018	7	Becerra-Tomas et al., 2019(81)	88,632	Spain, USA, Greece, Italy, United Kingdom	High vs. low	0·73 (0·62-0·86)	26%	0·23
Coronary heart disease mortality	May 7, 2018	6	Becerra-Tomas et al., 2019(81)	270,565	USA, United Kingdom, Australia, Sweden	High vs. low	0·73 (0·59-0·89)	63%	0·02
Myocardial infarction	June 2014	3	Grosso et al., 2017(79)	44,428	USA, Germany, Sweden	High vs. low	0·67 (0·54-0·83)	N.R.	N.R.
	May 7, 2018	2	Becerra-Tomas et al., 2019(81)	35,489	Sweden, USA, Denmark, Spain	High vs. low	0·73 (0·61-0·88)	0%	0·66
Total stroke±	June 2014	5	Grosso et al., 2017(79)	159,995	USA, Italy, Germany, Sweden	High vs. low	0·76 (0·60-0·96)	52%	N.R.
	August 2016	6	Rosato et al., 2017(80)	181,353	USA, China, the Netherlands, Greece, Italy, Australia	High vs. low	0·73 (0·59-0·91)	46%	0·10

	May 7, 2018	5	Becerra-Tomas et al., 2019(81)	79,287	Sweden, Hong Kong, USA, Greece, United Kingdom, Denmark, Spain	High vs. low	0·80 (0·71-0·90)	0%	0·63
Ischemic stroke	August 2016	5	Rosato et al., 2017(80)	206,562	USA, Sweden, Italy	High vs. low	0·82 (0·73-0·92)	0%	0·46
Hemorrhagic stroke	August 2016	4	Rosato et al., 2017(80)	203,994	USA, Sweden, Italy	High vs. low	1·01 (0·74-1·27)	35.6%	0·20
Stroke mortality	May 7, 2018	4	Becerra-Tomas et al., 2019(81)	195,644	Greece, USA, United Kingdom, Denmark, Sweden	High vs. low	0·87 (0·80-0·96)	0%	0·74
Type II diabetes	August 31, 2013	6	Esposito et al., 2014(68)	95,394	USA, Greece, Italy, Spain	High vs. low	0·80 (0·68-0·93)	63%	0·005
	April 2, 2014	9	Schwingshackl et al., 2015(82)	122,810	USA, United Kingdom, Spain, Greece, Denmark, France, Germany, Italy, Sweden, the Netherlands	High vs. low	0·81 (0·73-0·90)	55%	<0·0001
	December 31, 2015	6	Jannasch et al., 2017(63)	196,772	USA, Spain, Greece, Denmark, France, Germany, Italy, Sweden, United Kingdom, the Netherlands	High vs. low	0·87 (0·82-0·93)	26%	0·24
Cancer mortality	June 2017	6	Milajerdi et al., 2018(64)	789,104	USA	High vs. low	0·81 (0·78-0·83)	2%	0·420
Breast cancer	August 2016	5	Van den Brandt et al., 2017(83)	58,923	USA, United Kingdom, Sweden, the Netherlands, Denmark, France, Germany, Greece, Italy, Norway, Spain	High vs. low	0·94 (0·88-1.01)	13%	0·33
Gastric cancer	December 2018	2	Du et al., 2019(84)	956,518	USA, Denmark, United Kingdom, France, Sweden, Germany, Italy, Spain, the Netherlands, Norway, Greece	High vs. low	0·89 (0·68-1·17)	52%	0·10
Weight loss in adults	June 2010	12	Esposito et al., 2011(85)	2,683	Italy, USA, France, Israel, Greece, Spain, Germany, the Netherlands	MED diet vs. control diet	-1·75 (-2·86 to -0·64)	95%	0·001
	September 2018	13	Ge et al., 2020(73)	532	France, Luxembourg, Australia, the Netherlands, Spain, Italy, Greece, Algeria, Germany, Brazil, Sweden, USA, Poland, New Zealand, United Kingdom, Finland, Iceland, Denmark	MED diet vs. usual diet	-2·87 (-4·21 to 1·60)	N.R.	N.R.

Body mass index in adults	June 2010	15	Esposito et al., 2011(85)	3,337	Italy, USA, France, Israel, Greece, Spain, Germany	MED diet vs. control diet	-0·57 (-0·93 to -0·21)	92%	<0·001
Waist circumference in adults	February 9, 2016	29	Garcia et al., 2016(86)	4,133	Canada, Algeria, the Netherlands, Great Britain, Spain, Italy, USA, Greece, Chile, Sweden, Australia, Romania, South Africa	MED vs. control diet	-0·44 (-·48 to -0·41)	96%	<0·0001

Table S3 summarizes the findings of all meta-analyses identified.
Meta-analysis search date: the date of search reported by the meta-analysis.
Countries: the countries of included studies in the meta-analysis.
Unit of exposure: high versus low dietary metrics (categorical), point increase in score (continuous), or trial experimental and control groups.
RR: relative risk; 95% CI: 95% confidence interval.
DASH and all-cause mortality: Schwingshackl et al., 2015 considered a narrative review because <2 studies.
DASH and total stroke: Kontogianni et al., 2014 considered a narrative review because no quantitative meta-analysis reported for all studies.
DII and colorectal cancer: Steck et al., 2015 considered a narrative review because <2 studies.
HEI and diabetes: Schwingshackl et al., 2015 considered a narrative review because <2 studies.
HEI and esophageal cancer: Schwingshackl et al., 2018 considered a narrative review because <2 studies.
HEI and gastric cancer: Schwingshackl et al., 2018 considered a narrative review because <2 studies.
HEI and pancreatic cancer: Schwingshackl et al., 2018 considered a narrative review because <2 studies.
MED and myocardial infarction mortality: Becerra-Tomas et al., 2019 considered a narrative review because <2 studies.
MED and total stroke: Kontoganni et al., 2014 considered a narrative review because no quantitative meta-analysis reported for all individual studies.
MED and colorectal cancer: Steck et al., 2015 considered a narrative review because no quantitative meta-analysis reported for all studies.
AHEI: Alternative Healthy Eating Index; DASH: Dietary Approaches to Stop Hypertension; DII: Dietary Inflammatory Index; Healthy Eating Index; MED: Mediterranean Diet Score.
N.R.: not reported.
α Odds ratio and 95% CI reported.
± Unspecified stroke considered total stroke.
Unit of exposure for weight loss=kg, waist circumference=cm, body mass index=kg/m^2.

Foods and Nutrients included in the Dietary Metrics

DDS: dairy categorised as dairy products; grain categorised as grains/roots/tubers; meat categorised meat/meat products.

FCS: main staples categorised as grains/roots/tubers; pulses categorised as legumes; milk categorised as dairy products; sugar categorised as sugar/sweets/ candy.

HDDS FANTA: cereals categorised as refined grains; dairy categorised as dairy products; sweets categorised as sugar/sweets/candy.

IYCMDD: dairy categorised as dairy products; flesh foods (meat, fish, poultry and liver/organ meats) categorised as meat; other fruits and vegetables (fruits and vegetables excluding vitamin A-rich fruits and vegetables) categorised as fruits/vegetables.

MDD-W: grains, white roots and tubers categorised as grains/roots/tubers; dairy categorised as dairy products; meat, poultry and fish categorised as meat and fish; other vitamin A-rich fruits and vegetables categorised as vitamin A-rich fruits/ vegetables.

WDDS-IDDS (9 groupings): starchy staples categorised as grains/roots/ tubers; milk and milk products categorised as dairy products; organ meat categorised as meat; other vitamin-A rich fruits and other fruits and vegetables categorised as fruits/vegetables.

AHEI: whole grains categorised as grains/roots/ tubers; red and processed meat categorised as meat; long-chain (n-3) fats (EPA + DHA) and polyunsaturated fats categorised as polyunsaturated fat.

DASH: whole grains categorised as grains/roots/tubers; low-fat dairy products categorised as dairy products; red and processed meats categorised as meat.

DGAI: grain and whole grains categorised as grains/roots/tubers; starchy vegetable categorised as roots/tubers; milk and milk products categorised dairy products; meat and legume categorised as meat; orange vegetable and other vegetables categorised as vegetables, discretionary energy categorised as empty calories; variety of fruits and vegetables, and low-fat choices not categorised.

DII: garlic, ginger, onion categorised as vegetables; saffron, turmeric, pepper, thyme/oregano, rosemary categorised as spices/condiments/beverages; polyunsaturated fat, n-3 fatty acids, n-6 fatty acids categorised as polyunsaturated fat; other components not shown in table are: β-carotene, caffeine, energy, eugenol, saffron, turmeric, green/black tea, falvan-3-ol, flavones, flavonones, anthocyanidins, isoflavones, pepper, thyme/ oregano, rosemary DQI: grains categorised as grains/roots/tubers; dairy categorised as dairy products; meat and poultry categorised as meat; variety food groups not shown in Figure 1.

HEI: grains categorised as grains/roots/tubers; dairy categorised as dairy products; total fruit (includes fruit juice) and whole fruit (includes all forms except juice) categorised as fruits; greens and beans and total vegetables categorised as vegetables; whole PUFAs + MUFAs: SFAs

categorised as fatty acids ratio; empty calories (energy from solid fats, alcohol and added sugar) categorised as empty calorie foods.

KIDMED: pasta or rice, cereals or grains categorised as grains/roots/tubers; dairy foods for breakfast and greater than 2 daily servings of dairy products categorised as dairy products; daily fruit or fruit juice and eats second daily serving of fruit categorised as fruits; daily fresh or cooked vegetable serving and eats greater than 1 daily serving of fresh or cooked vegetables categorised as vegetables; olive oil categorised as oils/fats; commercial baked goods and pastries, and sweets and candy categorised as sugar/sweets/candy.

MED: cereals categorised as grains/roots/tubers; dairy categorised as dairy products; fruits and nuts categorised as fruits; ratio of MUFA:SFA categorised as fatty acids ratio.

PURE diet score: dairy categorised as dairy products; unprocessed red meat categorised as meat.

RFS: dark breads (whole wheat, rye, pumpernickel), cornbread/tortillas/grits, high-fiber cereals (bran, granola, shredded wheat), cooked cereals categorised as grains/roots/tubers; sweet potatoes/yams, other potatoes categorised as roots/tubers; 2% milk and beverages and 1% or skim milk categorised as dairy products; chicken/turkey categorised as meat; apples/pears, oranges, cantaloupe, orange/grapefruit juice, grapefruit, other fruit juices categorised as fruits; dried beans categorised as legumes; tomatoes, broccoli, spinach, mustard/turnip/collard greens, carrots, mixed vegetables with carrots categorised as vegetables; green salad categorised as dark green leafy vegetables.

WHO-HDI 2015: free sugar categorised as mono- and disaccharides.

WCRF-AICR: foods of plant origin categorised as fruits/vegetables; red and processed meat categorised as meat; energy-dense foods and sugary drinks categorised as empty calorie foods.

FVS does not include specific foods or nutrients.

Supplementary Material References

1. Marshall S, Burrows T, Collins CE. Systematic review of diet quality indices and their associations with health-related outcomes in children and adolescents. Journal of human nutrition and dietetics : the official journal of the British Dietetic Association. 2014;27(6):577-98.
2. Rah JH, Akhter N, Semba RD, de Pee S, Bloem MW, Campbell AA, et al. Low dietary diversity is a predictor of child stunting in rural Bangladesh. European journal of clinical nutrition. 2010;64(12):1393-8.
3. Kennedy GL, Pedro MR, Seghieri C, Nantel G, Brouwer I. Dietary diversity score is a useful indicator of micronutrient intake in non-breast-feeding Filipino children. The Journal of nutrition. 2007;137(2):472-7.
4. Coates J, Rogers BL, Webb P, Maxwell D, Houser R, McDonald C. Diet diversity study. Boston, M.A.: Tufts University; 2007.
5. Wiesmann D, Bassett L, Benson T, Hoddinott J. Validation of the World Food Programme's food consumption score and alternativer indicators of household food security. Washington, D.C.: IFPRI; 2009.
6. WFP Vulnerability Analysis and Mapping. Food consumption analysis: calculation and use of the food consumption score in food security analysis. Rome, Italy: World Food Programme; 2008.
7. Saibul N, Shariff ZM, Lin KG, Kandiah M, Ghani NA, Rahman HA. Food variety score is associated with dual burden of malnutrition in Orang Asli (Malaysian indigenous peoples) households: implications for health promotion. Asia Pacific journal of clinical nutrition. 2009;18(3):412-22.
8. Darapheak C, Takano T, Kizuki M, Nakamura K, Seino K. Consumption of animal source foods and dietary diversity reduce stunting in children in Cambodia. International archives of medicine. 2013;6:29.
9. Swindale A, Bilinsky P. Household dietary diversity score (HDDS) for measurement of household food access: indicator guide. Washintong, D.C.: FHI 360/FANTA; 2006.
10. Kennedy G, Ballard T, Dop MC. Guidelines for measuring household and individual dietary diversity. Rome, Italy: FANTA; 2011.
11. Hoddinott J, Yohannes I. Dietary diversity as a housheold food security indicator. Washington, D.C.: FANTA; 2002.
12. Working Group on Infant and Young Child Feeding Indicators. Developing and validating simple indicators of dietary quality and energy intake of infants and young children in developing countries: summary of findings from analysis of 10 data sets. Washington, D.C. : FANTA; 2006.
13. Working Group on Infant and Young Child Feeding Indicators. Developing and validating simple indicators of infants and young children in developing countries: additional analysis of 10 data sets. Washington, D.C.: FANTA; 2007.
14. Arimond M, Wiesmann D, Becquey E, Carriquiry A, Daniels MC, Deitchler M, et al. Simple food group diversity indicators predict micronutrient adequacy of women's diets in 5 diverse, resource-poor settings. Journal of Nutrition. 2010;140(11):2059S-69S.
15. Martin-Prevel Y, Allemand P, Wiesmann D, Arimond M, Ballard T, Dietchler M, et al. Moving forward on choosing a standard operational indicator of women's dietary diversity. Rome, Italy: FAO; 2015.

16. Fung TT, Chiuve SE, McCullough ML, Rexrode KM, Logroscino G, Hu FB. Adherence to a DASH-style diet and risk of coronary heart disease and stroke in women.[Erratum appears in Arch Intern Med. 2008 Jun 23;168(12):1276]. Arch Intern Med. 2008;168(7):713-20.
17. Fogli-Cawley JJ, Dwyer JT, Saltzman E, McCullough ML, Troy LM, Jacques PF. The 2005 Dietary Guidelines for Americans Adherence Index: development and application. Journal of Nutrition. 2006;136(11):2908-15.
18. Fogli-Cawley JJ, Dwyer JT, Saltzman E, McCullough ML, Troy LM, Meigs JB, et al. The 2005 Dietary Guidelines for Americans and risk of the metabolic syndrome. The American journal of clinical nutrition. 2007;86(4):1193-201.
19. Fogli-Cawley JJ, Dwyer JT, Saltzman E, McCullough ML, Troy LM, Meigs JB, et al. The 2005 Dietary Guidelines for Americans and insulin resistance in the Framingham Offspring Cohort. Diabetes care. 2007;30(4):817-22.
20. Mohseni-Takalloo S, Hosseini-Esfahani F, Mirmiran P, Azizi F. Associations of Pre-Defined Dietary Patterns with Obesity Associated Phenotypes in Tehranian Adolescents. Nutrients. 2016;8(8).
21. Hosseini-Esfahani F, Jessri M, Mirmiran P, Bastan S, Azizi F. Adherence to dietary recommendations and risk of metabolic syndrome: Tehran Lipid and Glucose Study. Metabolism: clinical and experimental. 2010;59(12):1833-42.
22. Jessri M, Lou WY, L'Abbe MR. The 2015 Dietary Guidelines for Americans is associated with a more nutrient-dense diet and a lower risk of obesity. American Journal of Clinical Nutrition. 2016;104(5):1378-92.
23. Shivappa N, Steck SE, Hurley TG, Hussey JR, Hebert JR. Designing and developing a literature-derived, population-based dietary inflammatory index. Public health nutrition. 2014;17(8):1689-96.
24. Shivappa N, Steck SE, Hurley TG, Hussey JR, Ma Y, Ockene IS, et al. A population-based dietary inflammatory index predicts levels of C-reactive protein in the Seasonal Variation of Blood Cholesterol Study (SEASONS). Public health nutrition. 2014;17(8):1825-33.
25. Tabung FK, Steck SE, Zhang J, Ma Y, Liese AD, Agalliu I, et al. Construct validation of the dietary inflammatory index among postmenopausal women. Annals of epidemiology. 2015;25(6):398-405.
26. Kim S, Haines PS, Siega-Riz AM, Popkin BM. The Diet Quality Index-International (DQI-I) provides an effective tool for cross-national comparison of diet quality as illustrated by China and the United States. Journal of Nutrition. 2003;133(11):3476-84.
27. Tur JA, Romaguera D, Pons A. The Diet Quality Index-International (DQI-I): is it a useful tool to evaluate the quality of the Mediterranean diet? British Journal of Nutrition. 2005;93(3):369-76.
28. Shin MK, Kim YS, Kim JH, Kim SH, Kim Y. Dietary Patterns and Their Associations with the Diet Quality Index-International (DQI-I) in Korean Women with Gestational Diabetes Mellitus. Clinical nutrition research. 2015;4(4):216-24.
29. Guenther PM, Reedy J, Krebs-Smith SM, Reeve BB. Evaluation of the Healthy Eating Index-2005. J Am Diet Assoc. 2008;108(11):1854-64.
30. Guenther PM, Reedy J, Krebs-Smith SM, Reeve BB, Basiotis PP. Development and evaluation of the healthy eating index-2005: technical report. Center for Nutrition Policy and Promotion, U.S. Department of Agriculture; 2007.

31. Guenther PM, Kirkpatrick SI, Reedy J, Krebs-Smith SM, Buckman DW, Dodd KW, et al. The Healthy Eating Index-2010 is a valid and reliable measure of diet quality according to the 2010 Dietary Guidelines for Americans. Journal of Nutrition. 2014;144(3):399-407.
32. Guenther PM, Casavale KO, Reedy J, Kirkpatrick SI, Hiza HA, Kuczynski KJ, et al. Update of the Healthy Eating Index: HEI-2010. Journal of the Academy of Nutrition and Dietetics. 2013;113(4):569-80.
33. Britten P, Cleveland LE, Koegel KL, Kuczynski KJ, Nickols-Richardson SM. Updated US Department of Agriculture Food Patterns meet goals of the 2010 dietary guidelines. Journal of the Academy of Nutrition and Dietetics. 2012;112(10):1648-55.
34. Lazarou C, Panagiotakos DB, Matalas AL. Physical activity mediates the protective effect of the Mediterranean diet on children's obesity status: The CYKIDS study. Nutrition (Burbank, Los Angeles County, Calif). 2010;26(1):61-7.
35. Dura Trave T, Castroviejo Gandarias A. [Adherence to a Mediterranean diet in a college population]. Nutr Hosp. 2011;26(3):602-8.
36. Sahingoz SA, Sanlier N. Compliance with Mediterranean Diet Quality Index (KIDMED) and nutrition knowledge levels in adolescents. A case study from Turkey. Appetite. 2011;57(1):272-7.
37. Mariscal-Arcas M, Rivas A, Velasco J, Ortega M, Caballero AM, Olea-Serrano F. Evaluation of the Mediterranean Diet Quality Index (KIDMED) in children and adolescents in Southern Spain. Public health nutrition. 2009;12(9):1408-12.
38. Stefan L, Prosoli R, Juranko D, Cule M, Milinovic I, Novak D, et al. The Reliability of the Mediterranean Diet Quality Index (KIDMED) Questionnaire. Nutrients. 2017;9(4).
39. Serra-Majem L, Ribas L, Ngo J, Ortega RM, Garcia A, Perez-Rodrigo C, et al. Food, youth and the Mediterranean diet in Spain. Development of KIDMED, Mediterranean Diet Quality Index in children and adolescents. Public health nutrition. 2004;7(7):931-5.
40. Garcia Cabrera S, Herrera Fernandez N, Rodriguez Hernandez C, Nissensohn M, Roman-Vinas B, Serra-Majem L. KIDMED TEST; PREVALENCE OF LOW ADHERENCE TO THE MEDITERRANEAN DIET IN CHILDREN AND YOUNG; A SYSTEMATIC REVIEW. Nutr Hosp. 2015;32(6):2390-9.
41. Martin-Calvo N, Chavarro JE, Falbe J, Hu FB, Field AE. Adherence to the Mediterranean dietary pattern and BMI change among US adolescents. International journal of obesity (2005). 2016;40(7):1103-8.
42. Trichopoulou A, Costacou T, Bamia C, Trichopoulos D. Adherence to a Mediterranean diet and survival in a Greek population. N Engl J Med. 2003;348(26):2599-608.
43. Trichopoulou A, Kouris-Blazos A, Wahlqvist ML, Gnardellis C, Lagiou P, Polychronopoulos E, et al. Diet and overall survival in elderly people. Bmj. 1995;311(7018):1457-60.
44. Rumawas ME, Dwyer JT, McKeown NM, Meigs JB, Rogers G, Jacques PF. The development of the Mediterranean-style dietary pattern score and its application to the American diet in the Framingham Offspring Cohort. The Journal of nutrition. 2009;139(6):1150-6.
45. Buckland G, Gonzalez CA, Agudo A, Vilardell M, Berenguer A, Amiano P, et al. Adherence to the Mediterranean diet and risk of coronary heart disease in the Spanish EPIC Cohort Study. American journal of epidemiology. 2009;170(12):1518-29.
46. Mente A. Association of dietary quality and risk of cardiovascular disease and mortality in 218,000 people from over 50 countries. [Presentation]. In press 2018.

47. Kant AK, Schatzkin A, Graubard BI, Schairer C. A prospective study of diet quality and mortality in women. Jama. 2000;283(16):2109-15.
48. Collins CE, Burrows TL, Rollo ME, Boggess MM, Watson JF, Guest M, et al. The comparative validity and reproducibility of a diet quality index for adults: the Australian Recommended Food Score. Nutrients. 2015;7(2):785-98.
49. Burrows TL, Collins K, Watson J, Guest M, Boggess MM, Neve M, et al. Validity of the Australian Recommended Food Score as a diet quality index for Pre-schoolers. Nutrition journal. 2014;13:87.
50. Marshall S, Watson J, Burrows T, Guest M, Collins CE. The development and evaluation of the Australian child and adolescent recommended food score: a cross-sectional study. Nutrition journal. 2012;11:96.
51. Kaluza J, Hakansson N, Brzozowska A, Wolk A. Diet quality and mortality: a population-based prospective study of men. European journal of clinical nutrition. 2009;63(4):451-7.
52. Huijbregts P, Feskens E, Räsänen L, Fidanza F, Nissinen A, Menotti A, et al. Dietary pattern and 20 year mortality in elderly men in Finland, Italy, and the Netherlands: longitudinal cohort study. Bmj. 1997;315(7099):13-7.
53. Stefler D, Pikhart H, Jankovic N, Kubinova R, Pajak A, Malyutina S, et al. Healthy diet indicator and mortality in Eastern European populations: prospective evidence from the HAPIEE cohort. European journal of clinical nutrition. 2014;68:1346.
54. Jankovic N, Geelen A, Streppel MT, de Groot LC, Kiefte-de Jong JC, Orfanos P, et al. WHO guidelines for a healthy diet and mortality from cardiovascular disease in European and American elderly: the CHANCES project. The American journal of clinical nutrition. 2015;102(4):745-56.
55. Jennings A, Welch A, van Sluijs EM, Griffin SJ, Cassidy A. Diet quality is independently associated with weight status in children aged 9-10 years. The Journal of nutrition. 2011;141(3):453-9.
56. Haveman-Nies A, Tucker KL, de Groot LC, Wilson PW, van Staveren WA. Evaluation of dietary quality in relationship to nutritional and lifestyle factors in elderly people of the US Framingham Heart Study and the European SENECA study. European journal of clinical nutrition. 2001;55(10):870-80.
57. Jankovic N, Geelen A, Winkels RM, Mwungura B, Fedirko V, Jenab M, et al. Adherence to the WCRF/AICR Dietary Recommendations for Cancer Prevention and Risk of Cancer in Elderly from Europe and the United States: A Meta-Analysis within the CHANCES Project. Cancer epidemiology, biomarkers & prevention : a publication of the American Association for Cancer Research, cosponsored by the American Society of Preventive Oncology. 2017;26(1):136-44.
58. Romaguera D, Vergnaud AC, Peeters PH, van Gils CH, Chan DS, Ferrari P, et al. Is concordance with World Cancer Research Fund/American Institute for Cancer Research guidelines for cancer prevention related to subsequent risk of cancer? Results from the EPIC study. The American journal of clinical nutrition. 2012;96(1):150-63.
59. Vergnaud AC, Romaguera D, Peeters PH, van Gils CH, Chan DS, Romieu I, et al. Adherence to the World Cancer Research Fund/American Institute for Cancer Research guidelines and risk of death in Europe: results from the European Prospective Investigation into Nutrition and Cancer cohort study1,4. The American journal of clinical nutrition. 2013;97(5):1107-20.

60. Schwingshackl L, Hoffmann G. Diet quality as assessed by the Healthy Eating Index, the Alternate Healthy Eating Index, the Dietary Approaches to Stop Hypertension score, and health outcomes: a systematic review and meta-analysis of cohort studies. Journal of the Academy of Nutrition and Dietetics. 2015;115(5):780-800.e5.
61. Onvani S, Haghighatdoost F, Surkan PJ, Larijani B, Azadbakht L. Adherence to the Healthy Eating Index and Alternative Healthy Eating Index dietary patterns and mortality from all causes, cardiovascular disease and cancer: a meta-analysis of observational studies. Journal of human nutrition and dietetics : the official journal of the British Dietetic Association. 2017;30(2):216-26.
62. Schwingshackl L, Bogensberger B, Hoffmann G. Diet Quality as Assessed by the Healthy Eating Index, Alternate Healthy Eating Index, Dietary Approaches to Stop Hypertension Score, and Health Outcomes: An Updated Systematic Review and Meta-Analysis of Cohort Studies. Journal of the Academy of Nutrition and Dietetics. 2018;118(1):74-100.e11.
63. Jannasch F, Kroger J, Schulze MB. Dietary Patterns and Type 2 Diabetes: A Systematic Literature Review and Meta-Analysis of Prospective Studies. The Journal of nutrition. 2017;147(6):1174-82.
64. Milajerdi A, Namazi N, Larijani B, Azadbakht L. The Association of Dietary Quality Indices and Cancer Mortality: A Systematic Review and Meta-analysis of Cohort Studies. Nutrition and cancer. 2018;70(7):1091-105.
65. Salehi-Abargouei A, Maghsoudi Z, Shirani F, Azadbakht L. Effects of Dietary Approaches to Stop Hypertension (DASH)-style diet on fatal or nonfatal cardiovascular diseases--incidence: a systematic review and meta-analysis on observational prospective studies. Nutrition. 2013;29(4):611-8.
66. Yang ZQ, Yang Z, Duan ML. Dietary approach to stop hypertension diet and risk of coronary artery disease: a meta-analysis of prospective cohort studies. International journal of food sciences and nutrition. 2019;70(6):668-74.
67. Feng Q, Fan S, Wu Y, Zhou D, Zhao R, Liu M, et al. Adherence to the dietary approaches to stop hypertension diet and risk of stroke: A meta-analysis of prospective studies. Medicine. 2018;97(38):e12450.
68. Esposito K, Chiodini P, Maiorino MI, Bellastella G, Panagiotakos D, Giugliano D. Which diet for prevention of type 2 diabetes? A meta-analysis of prospective studies. Endocrine. 2014;47(1):107-16.
69. Ali Mohsenpour M, Fallah-Moshkani R, Ghiasvand R, Khosravi-Boroujeni H, Mehdi Ahmadi S, Brauer P, et al. Adherence to Dietary Approaches to Stop Hypertension (DASH)-Style Diet and the Risk of Cancer: A Systematic Review and Meta-Analysis of Cohort Studies. Journal of the American College of Nutrition. 2019;38(6):513-25.
70. Mohseni R, Mohseni F, Alizadeh S, Abbasi S. The Association of Dietary Approaches to Stop Hypertension (DASH) Diet with the Risk of Colorectal Cancer: A Meta-Analysis of Observational Studies. Nutrition and cancer. 2020;72(5):778-90.
71. Tangestani H, Salari-Moghaddam A, Ghalandari H, Emamat H. Adherence to the Dietary Approaches to Stop Hypertension (DASH) dietary pattern reduces the risk of colorectal cancer: A systematic review and meta-analysis. Clinical nutrition (Edinburgh, Scotland). 2020.
72. Soltani S, Shirani F, Chitsazi MJ, Salehi-Abargouei A. The effect of dietary approaches to stop hypertension (DASH) diet on weight and body composition in adults: a systematic review and meta-analysis of randomized controlled clinical trials. Obesity reviews : an official journal of the International Association for the Study of Obesity. 2016;17(5):442-54.

73. Ge L, Sadeghirad B, Ball GDC, da Costa BR, Hitchcock CL, Svendrovski A, et al. Comparison of dietary macronutrient patterns of 14 popular named dietary programmes for weight and cardiovascular risk factor reduction in adults: systematic review and network meta-analysis of randomised trials. Bmj. 2020;369:m696.
74. Shivappa N, Hebert JR, Kivimaki M, Akbaraly T. Alternate Healthy Eating Index 2010, Dietary Inflammatory Index and risk of mortality: results from the Whitehall II cohort study and meta-analysis of previous Dietary Inflammatory Index and mortality studies. The British journal of nutrition. 2017;118(3):210-21.
75. Zahedi H, Djalalinia S, Sadeghi O, Asayesh H, Noroozi M, Gorabi AM, et al. Dietary Inflammatory Potential Score and Risk of Breast Cancer: Systematic Review and Meta-analysis. Clinical breast cancer. 2018;18(4):e561-e70.
76. Du M, Liu SH, Mitchell C, Fung TT. Associations between Diet Quality Scores and Risk of Postmenopausal Estrogen Receptor-Negative Breast Cancer: A Systematic Review. The Journal of nutrition. 2018;148(1):100-8.
77. Berhe K, Kidanemariam A, Gebremariam G, Gebremariam A. Prevalence and associated factors of adolescent undernutrition in Ethiopia: a systematic review and meta-analysis. BMC nutrition. 2019;5:49.
78. Bonaccio M, Di Castelnuovo A, Costanzo S, Gialluisi A, Persichillo M, Cerletti C, et al. Mediterranean diet and mortality in the elderly: a prospective cohort study and a meta-analysis. The British journal of nutrition. 2018;120(8):841-54.
79. Grosso G, Marventano S, Yang J, Micek A, Pajak A, Scalfi L, et al. A comprehensive meta-analysis on evidence of Mediterranean diet and cardiovascular disease: Are individual components equal? Crit Rev Food Sci Nutr. 2017;57(15):3218-32.
80. Rosato V, Temple NJ, La Vecchia C, Castellan G, Tavani A, Guercio V. Mediterranean diet and cardiovascular disease: a systematic review and meta-analysis of observational studies. European journal of nutrition. 2017.
81. Becerra-Tomas N, Blanco Mejia S, Viguiliouk E, Khan T, Kendall CWC, Kahleova H, et al. Mediterranean diet, cardiovascular disease and mortality in diabetes: A systematic review and meta-analysis of prospective cohort studies and randomized clinical trials. Crit Rev Food Sci Nutr. 2020;60(7):1207-27.
82. Schwingshackl L, Missbach B, Konig J, Hoffmann G. Adherence to a Mediterranean diet and risk of diabetes: a systematic review and meta-analysis. Public health nutrition. 2015;18(7):1292-9.
83. van den Brandt PA, Schulpen M. Mediterranean diet adherence and risk of postmenopausal breast cancer: results of a cohort study and meta-analysis. International journal of cancer. 2017;140(10):2220-31.
84. Du S, Li Y, Su Z, Shi X, Johnson NL, Li P, et al. Index-based dietary patterns in relation to gastric cancer risk: a systematic review and meta-analysis. The British journal of nutrition. 2019:1-11.
85. Esposito K, Kastorini CM, Panagiotakos DB, Giugliano D. Mediterranean diet and weight loss: meta-analysis of randomized controlled trials. Metabolic syndrome and related disorders. 2011;9(1):1-12.
86. Garcia M, Bihuniak JD, Shook J, Kenny A, Kerstetter J, Huedo-Medina TB. The Effect of the Traditional Mediterranean-Style Diet on Metabolic Risk Factors: A Meta-Analysis. Nutrients. 2016;8(3):168.
